# Design, synthesis, and biological evaluation of new thieno[2,3-*d*] pyrimidine derivatives as targeted therapy for PI3K with molecular modelling study

**DOI:** 10.1080/14756366.2021.2010729

**Published:** 2021-12-26

**Authors:** Fatma M. Elmenier, Deena S. Lasheen, Khaled A. M. Abouzid

**Affiliations:** a Faculty of Pharmacy, Pharmaceutical Chemistry Department, Ain Shams University, Cairo, Egypt; b Faculty of Pharmacy, Department of Organic and Medicinal Chemistry, University of Sadat City, Menoufia, Egypt

**Keywords:** Thieno[2,3-*d*] pyrimidine, PI3K and its isoforms, lipid kinase, cancer, molecular docking

## Abstract

Cancer is one of the most aggressive diseases characterised by abnormal growth and uncontrolled cell division. PI3K is a lipid kinase involved in cancer progression which makes it fruitful target for cancer control. 28 new morpholine based thieno[2,3-*d*] pyrimidine derivatives were designed and synthesised as anti-PI3K agents maintaining the common pharmacophoric features of several potent PI3K inhibitors. Their antiproliferative activity on NCI 60 cell lines as well as their enzymatic activity against PI3K isoforms were evaluated. Three compounds revealed good cytotoxic activities against breast cancer cell lines, especially T-47D. Compound **VIb** exhibited the best enzymatic inhibitory activity (72% & 84% on PI3Kβ & PI3Kγ), respectively and good activity on most NCI cell lines especially those with over expressed PI3K. Docking was carried out into PI3K active site which showed comparable binding mode to that of the PI-103 inhibitor. Compound **VIb** could be optimised to serve as a new chemical entity for discovering new anticancer agents.

## Graphical Abstract

**Figure:**
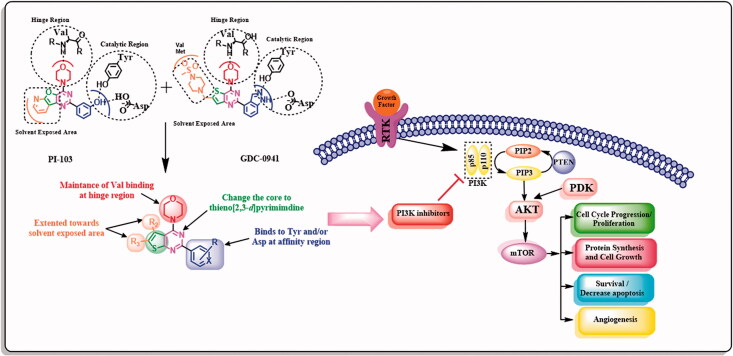

## 1. Introduction

Cancer could regenerate itself due to its ability to proliferate indefinitely by maintaining reproductive signals or overexpression of growth factors. It may also be initiated through abnormal activation of downstream signalling pathways, especially phosphatidyl inositol 3-kinase (PI3K)/the mammalian target of rapamycin (m-TOR) pathway or abnormal inactivation of suppressor genes^1–5^.

Phosphatidyl inositol 3-kinase (PI3K) is a lipid kinase crucial in a signal transduction pathway^6–9^. Dysregulation of PI3K pathway has also been observed in numerous pathologies including diabetes, thrombosis, rheumatoid arthritis, asthma as well as cancer^10^. PI3K phosphorylates the 3′-hydroxy position of the inositol ring as a result of growth factors or G-protein coupled receptors (GPCRs) activation, to give the second messenger phosphatidylinositol-3,4,5-trisphosphate (PIP3), that activates another protein kinase B (AKT) as well as other cellular messengers like 3-phosphoinositide-dependent protein kinase(PDK)^11–12^. Activation of this pathway promotes cell survival and growth and angiogenesis as well as inhibits apoptosis through various pathways^13–16^.

**Figure 1.**
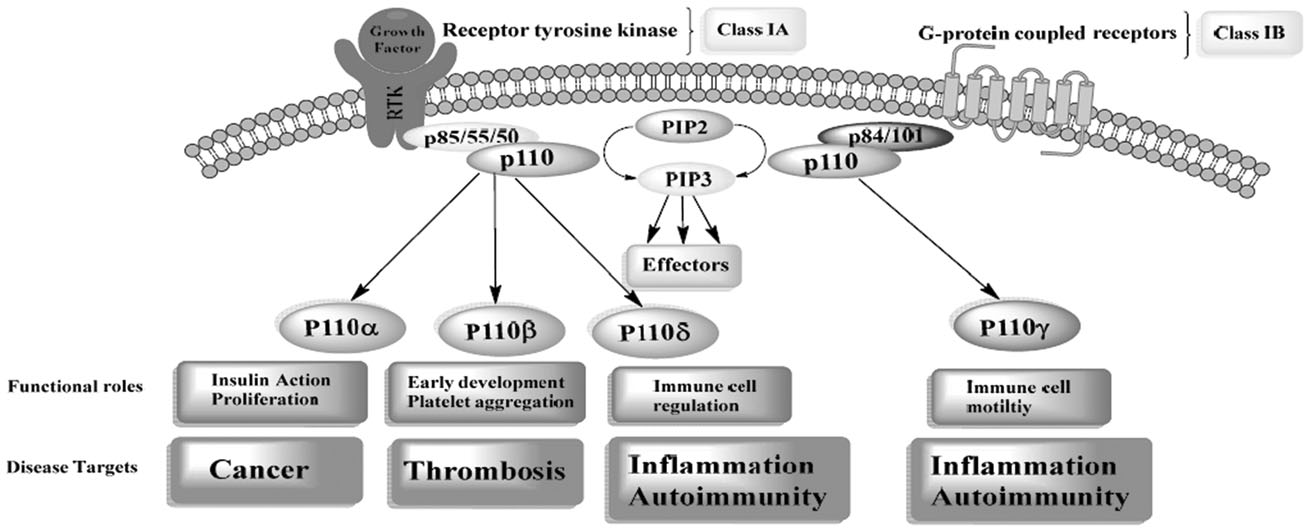
Summary of the function roles and disease targets associated with class I PI3K.

In mammals, PI3K family can be divided into 3 main classes (Class I, II, and III) depending on their structures and substrate specificities. Class I PI3Ks are sub-divided into two subclasses; IA and IB. Class IA PI3K members are heterodimers that consist of a catalytic subunit either (p110α, p110β or p110*δ* isoforms) associated with one of the five adaptor/regulatory subunit isoforms(p85), p85α (and its relevant variants p55α and p50α), p85β and p55γ which mediates PI3K activity^17–18^. Class IB PI3K enzymes are heterodimers that consist of a p110γ as catalytic subunit associated with p101 or p87 as the regulatory isoforms^17^. Although class II PI3K members are monomeric lipid kinases, class III PI3K enzymes are heterodimers that consist of a catalytic subunit and a regulatory subunit. The roles and the potential disease targets of class I PI3K can be summarised in Figure 1.

**Figure 2.**
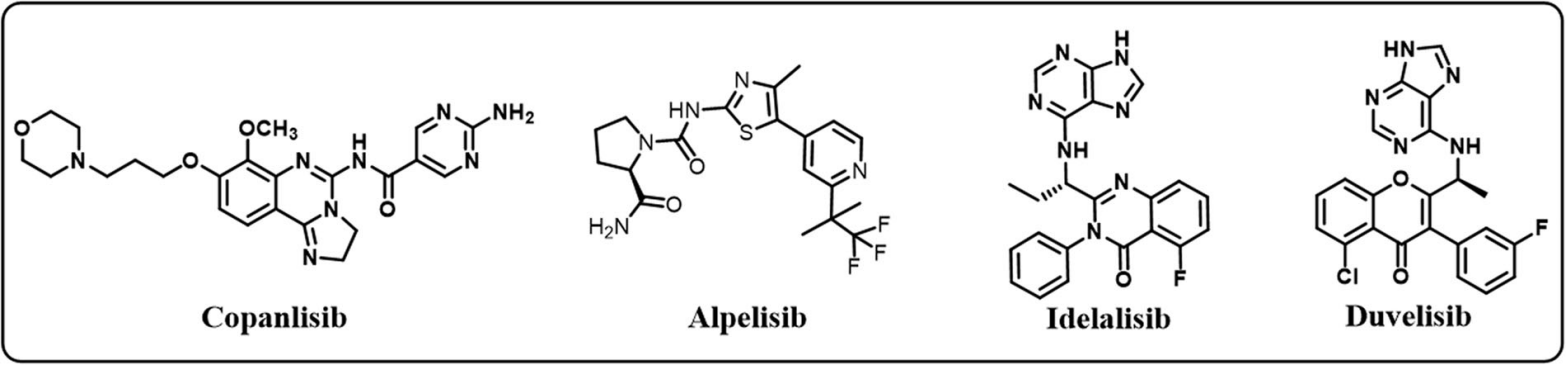
Different FDA approved (PI3K) inhibitors.

Based on the previous illustration, PI3K/mTOR pathway can be considered a fruitful target to design new targeted anticancer agents. The inhibitors can be classified according to selectivity to three main classes: pan-PI3K inhibitors, selective isoform PI3K inhibitors, and dual PI3K/mTOR inhibitors. There is another classification according to structure: morpholine-based PI3K inhibitors and non-morpholine-based PI3K inhibitors^16^ (Figure 2).

For example, Copanlisib is a non-morpholine-based pan PI3K inhibitor that was FDA approved in 2017^19^. Alpelisib is an orally bioactive non-morpholine-based PI3Kα inhibitor FDA approved in 2019^20^. Idelalisib, also known as Zydelig, was FDA approved in 2014 as a selective PI3K*δ* inhibitor that treats various CLL types^21^. Duvelisib is a potent non-morpholine selective dual PI3K*δ*/γ inhibitor that was approved by FDA in September 2018^22–26^.

## 2. Rationale and design

**Figure 3.**
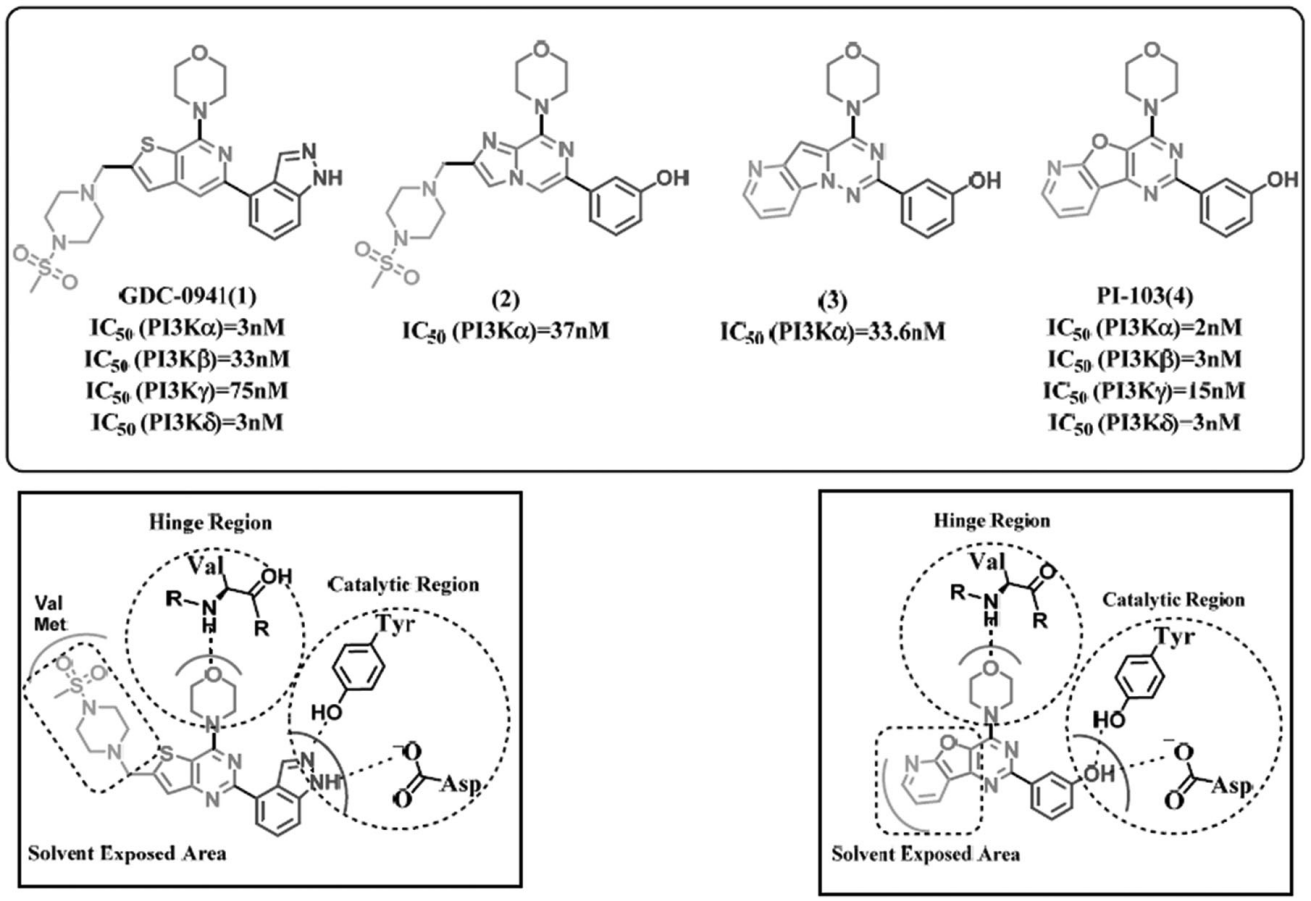
Several reported potent morpholine based PI3K inhibitors with examples of binding mode.

The common pharmacophoric features of several potent PI3K inhibitors (Figure 3) are summarised as follows: the morpholine ring is crucial for binding to Val amino acid (Val851, Val848, and Val882 in PI3Kα, PI3Kβ, and PI3Kγ, respectively) at hinge region (colored red). The central core can be a heterocycle (either fused or single) having an aryl substituent at meta-position to morpholine moiety (colored violet and green). HB donor/acceptor group should be present on the aryl substituent preferably at 3 positions to maintain the same HBs with key amino acids (Tyr, Asp and/or Lys) at affinity region (Tyr836, Asp810 or Lys802 for PI3Kα) (Tyr833, Asp807 for PI3Kβ) (Tyr867, Asp841 and Lys833 for PI3Kγ) (colored blue). Some derivatives are extended towards the solvent-exposed area in PI3K binding site. Hence, form additional interactions with surrounding amino acids or improve the pharmacokinetics of the designed compounds (colored orange). Although the most important regions in the binding site can be described as 4 main regions (hinge region, specificity region, affinity region, and non-conserved region), there are still unclear key aspects to inhibitor selectivity such as the exact contribution of the specificity pocket, the way by which hinge and affinity binding motifs affects selectivity, and the influence of conserved regions. In general, isoform selectivity and inhibitor binding result from a complex combination of interactions throughout the binding site, affected by protein and inhibitor conformational flexibility^27^.

The crystal structure of PI-103 (4) with PI3Kα (PDB: 4L23) revealed that it adopts a flat conformation and sits between Val851, Tyr836 and Asp810 on one side and Met922, Ile932, and Asp933 on the other side where it forms three main HBs with the binding site as follows; The morpholine oxygen makes HB with the hinge Val851. The phenolic OH makes two HBs with the carboxyl group of Asp810 and the hydroxyl group of Tyr836. Structurally, Met772 exists in the flexible loop of PI3K which takes “down” conformation during binding of PI-103(4) with the active site. Also binding of PIP-103(4) induces a conformation change of the Lys802 side chain which leads to the vast space which can accommodate various large substituents in the cavity and provide a potential direction to design more potent and selectivity inhibitors against PI3K^28^.

**Figure 4.**
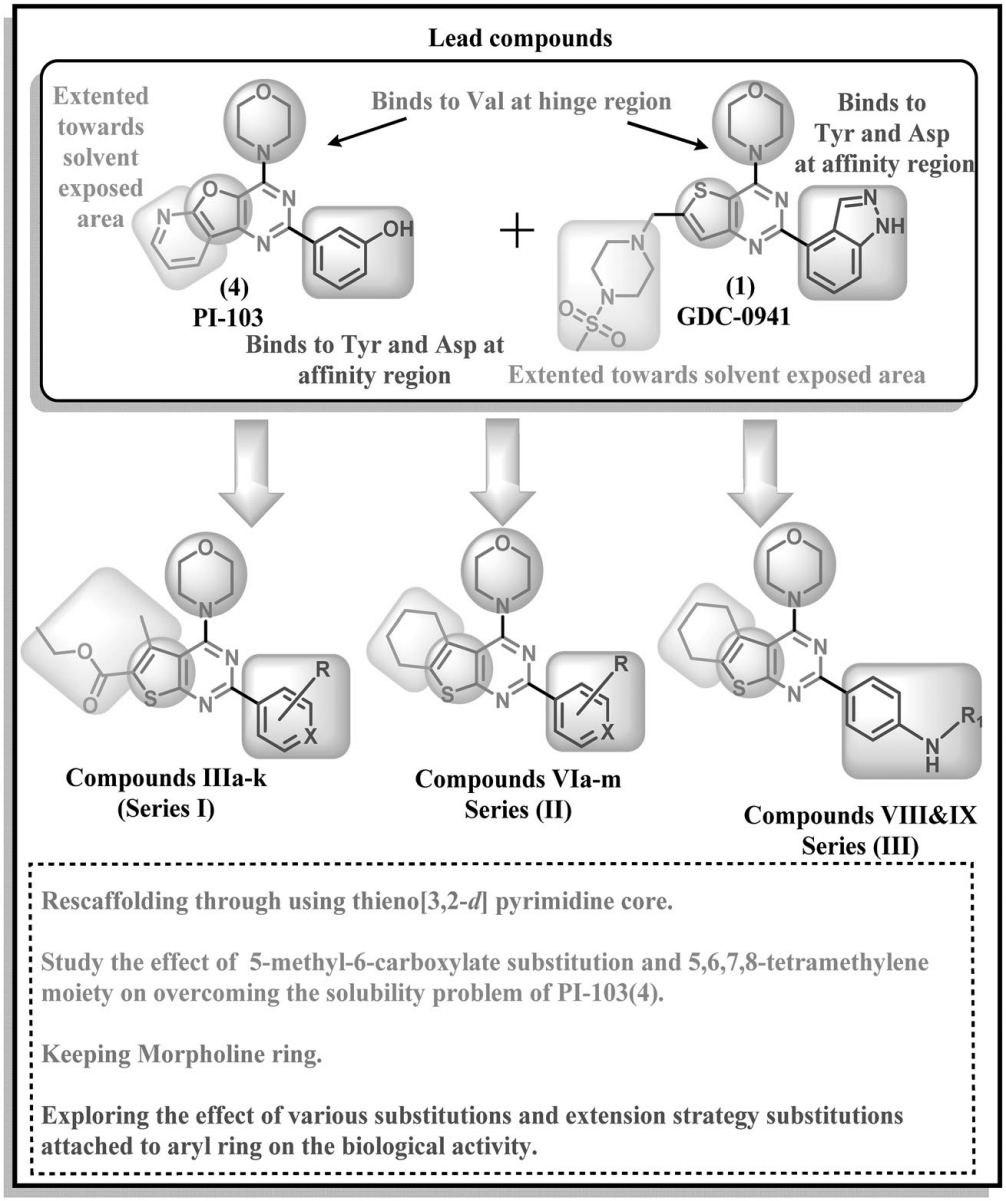
Design of proposed new PI3K inhibitors by structural modification of the lead compound PI-103.

Based on the previously mentioned SAR, a novel series of fused pyrimidine derivatives targeting PI3K enzyme was designed, synthesised, and evaluated. The design strategy was based on maintaining the morpholine moiety in the correct orientation similar to that of the lead compound while modifying the main scaffold aiming to overcome the poor pharmacokinetics and solubility problems in PI-103(4). Meanwhile, exploring the effect of various substitutions on the aryl ring as well as attempting extension strategy on phenyl ring as illustrated in Figure 4.

## 3. Results and discussion

### 3.1. Chemistry

**Scheme 1.**
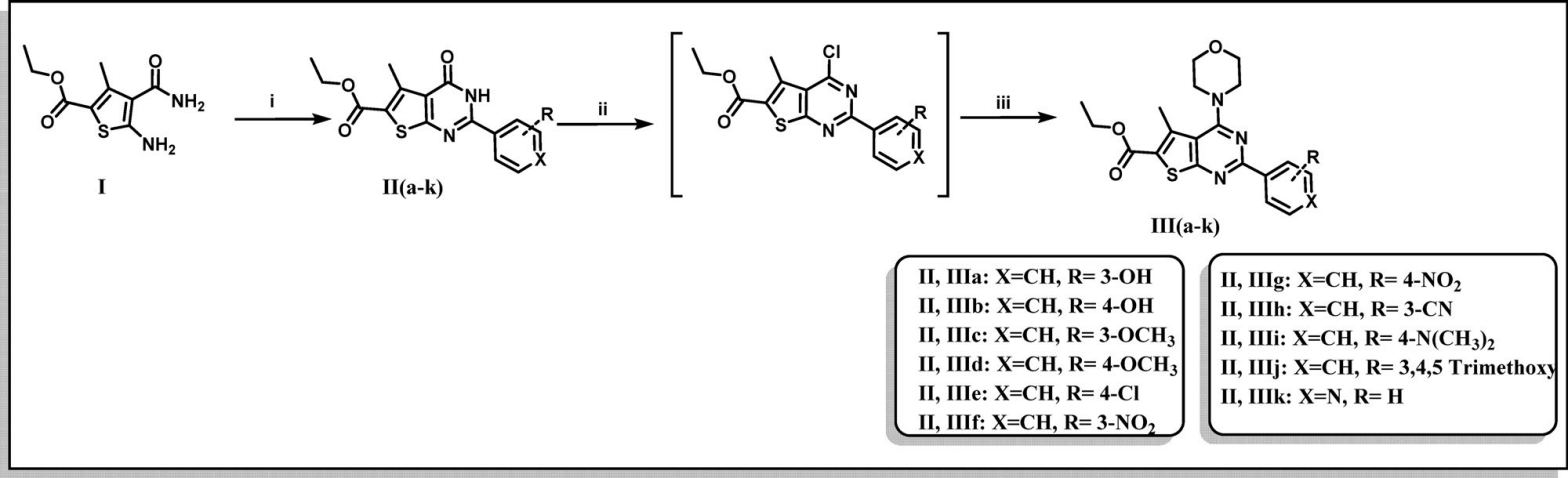
Preparation of ethyl 2-aryl-5-methyl-4-morpholino- thieno[2,3-d]pyrimidine-6-carboxylate (**IIIa–k**). Reagents & Conditions: (i) Substituted aldehydes, HCl, DMF, reflux, overnight, 60–68%, (ii) POCl_3_, reflux, 6 h, 50–70%, (iii) Morpholine, absolute ethanol: absolute isopropanol (1:1), reflux, 4 h, 75–85%.

**Scheme 2.**
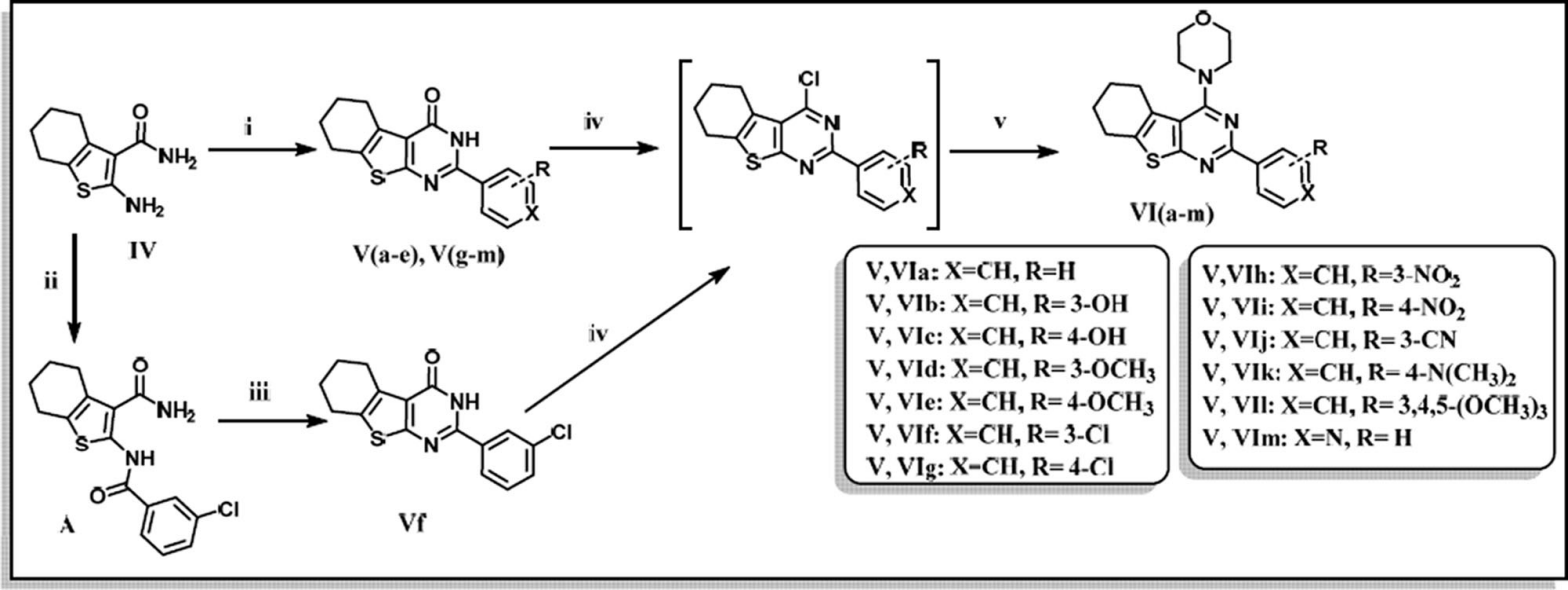
Preparation of 2-aryl-4-morpholino-5,6,7,8-tetrahydrobenzo[4,5]thieno[2,3-d]pyrimidine (**Va–m**). Reagents & Conditions: (i) substituted aldehydes, HCl, DMF, reflux, overnight, 70–85% (ii) 3-chlorobenzoyl chloride, pyridine, stir, RT, overnight, 75% (iii) 2 N NaOH, ethanol, reflux, 24 h, 50% (iv) POCl_3_, reflux, 6 h, 75–85% (v) Morpholine, absolute ethanol: absolute isopropanol (1:1), reflux, 4 h to overnight, 75–95%.

**Scheme 3.**
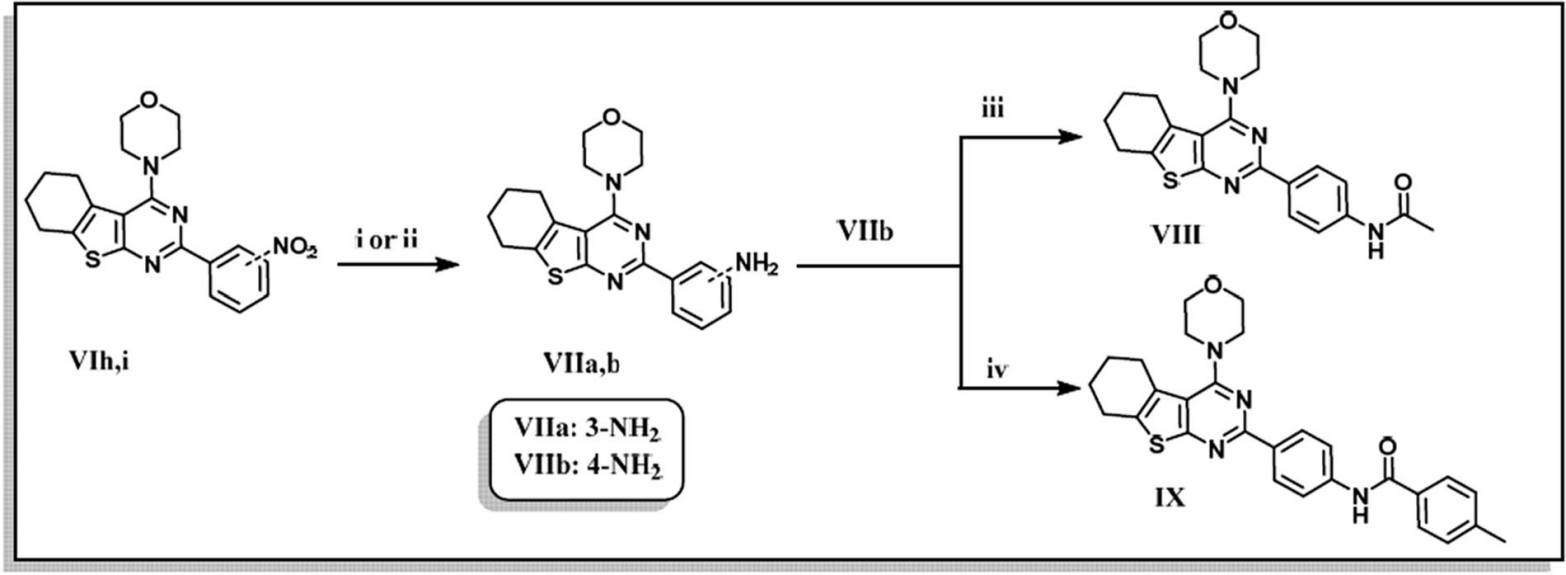
Preparation of 2-(4-anilino)-4-(4-morpholino)-5,6 substituted thienopyrimidine derivatives (**VII–IX**). Reagents & Conditions: (i) H_2_/Pd, DCM, 8 h, 90% (ii) Sodium dithionite,10% NaOH, acetone, reflux, 3 h, 85% (iii) Acetic anhydride, dry DCM, RT, 85% (iv) p-Tolyl chloride, TEA, Dry DCM, RT, overnight, 80%.

The designed thienopyrimidine based inhibitors (**IIIa–k, VIa–m, VIII**, and **IX**) were synthesised through the synthetic routes outlined in (Schemes 1–3). Beginning with “Gewald Aminothiophene Synthesis”^29^ to prepare the key 2-aminothiophene intermediates (**I** and **IV**) through a one-pot reaction involving ethyl acetoacetate or cyclohexanone, activated cyanoacetamide and elemental sulphur in presence morpholine as base in DMF as polar solvent^30–35^. Then thieno[2,3-*d*]pyrimidin-4-one derivatives **IIa–k** and **Va–e** and **Vg–m** were synthesised by reaction of **I** or **IV** with different aldehydes in presence of a catalytic amount of concentrated HCl and in dry DMF^36–38^. Except for compound **Vf**^39^, which was prepared via formation of 2-(3-chlorobenzamido)-4,5,6,7-tetrahydrobenzo[*b*]thiophene-3-carboxamide (**A**)^40^ followed by reflux with (2 N) sodium hydroxide in isopropanol^41–42^. Heating **IIa–k** and **Va–m** under reflux in neat POCl_3_^43–44^ afforded the chlorinated derivatives which were directly used in the next step without further purification. Nucleophilic substitution of the chloro derivative with morpholine in a mixture of ethanol/isopropanol (1:1)^45^ and TEA as base provided the final compounds **IIIa–k** and **VIa–m**. Compound **VIIa** was prepared by catalytic hydrogenation of compound **VIh** using (10% Pd/C)^46–48^, hydrogenator (Parr Shaker) as a source of hydrogen and DCM as solvent. However, compound **VIIb** was prepared by dissolving compound **VIi** in acetone and 0.5 N NaOH in presence of sodium dithionite^49^. Finally, compounds **VIII** and **IX** were obtained by acetylating **VIIb** with acetic anhydride and 4-methylbenzoyl chloride, respectively, in dry DCM^50^ as solvent and presence of TEA as a base in synthesis of **IX**.

### 3.2. Biological evaluation

**Figure 5.**
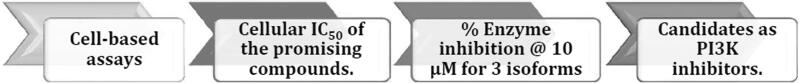
Sequential scenario for the development of PI3K inhibitors.

The development of PI3K inhibitors went through a sequential biological evaluation process involving cycles of cell-based and measuring cellular IC_50_ for the most promising compounds and finally, enzymatic assays (Figure 5). First, 28 thienopyrimidine derivatives were screened through the cytotoxicity assay on 60 human cell lines, then cellular IC_50_ was determined on T-47D for the most promising three compounds. Finally, PI3K inhibitory activity was measured against the isolated isoforms of the PI3K enzyme at 10 μM.

#### 3.2.1. *In vitro* antiproliferative activity against NCI 60 cell line

All designed 28 compounds **IIIa–k**, **VIa–m**, **VIIa, b** and **VIII, IX** were selected by the Developmental Therapeutics Program (DTP) of the National Cancer Institute (NCI), division of cancer treatment and diagnosis, MD, USA (http://dtp.nci.nih.gov/), to be evaluated in the NCI-60 cell-line screen. Compounds were tested at an initial 1-dose (10 μM) assay determining the growth percentage of the full NCI-60 panel of human tumour cell lines, which represent different tumours including Leukaemia, NSCLC, Melanoma, Colon, CNS, Ovarian, Renal, Prostate, and Breast Cancers^51^.

Upon analysis of the NCI-60 results, the following observations could be outlined: compound **IIIa** having a meta hydroxyl moiety exhibited significant-good activity on ovarian, renal, and breast cancer cell lines (IGROV1, RXF-393, HS-578T, and T-47D), that mainly over expresses PI3K enzyme, with 83.1%, 81.6%, 84.5%, and 94.7% inhibition, respectively. Moreover, compound **IIIb** having a para hydroxyl moiety exhibited good activity on renal and breast cancer cell lines (UO-31 and T-47D) with 71.7% and 83.3% inhibition, respectively. Similarly, compound **VIb** having a meta hydroxyl moiety showed potent activity on NSCLC, CNS, ovarian, renal, and breast cancer cell lines (HOP-92, SNB-75, IGROV1, UO-31, and T-47D) with 88.8%, 92.9%, 96.1%, 86.9% and 90.8% inhibition, respectively. Unfortunately, Compound **VIc** having a para hydroxyl moiety showed significant inhibitory activity only on the breast cancer cell line (T-47D) with 76.9% inhibition.

Despite its poor enzymatic activity in biochemical assay, compound **IIIj** having 3,4,5, trimethoxy moieties showed the best mean cell line inhibitory activity of 66.9%, especially on NSCLC cell line HOP-92 (115%), melanoma cell line SK-MEL-5 (97.1%), and breast cancer cell line T-47D (106.9%) (antiproliferative activity against NCI 60 cell lines is provided in Tables S1, S2 and S3).

#### 3.2.2. *In vitro* cytotoxic activity assay against T-47D cancer cell line (IC_50_)

The cytotoxic activity of the most promising synthesised compounds (**IIIa**, **IIIj** and **VIb**) was also evaluated against the breast cancer (T-47D) cell line, by SRB assay (Routine Analysis) (0.01,0.1,1,10,100 μM). T-47D was chosen to perform further cytotoxic activity as it is a tumour cell with the PIK3CA mutation^52^. PIK3CA gene responses for making (p110α) protein which is a subunit of PI3K^53^. The PIK3CA mutation overexpresses in breast cancer^54–57^. T-47D is a Breast Ductal Carcinoma cell line was obtained from Nawah Scientific Inc., (Mokatam, Cairo, Egypt). Cells were maintained in DMEM media supplemented with 100 mg/ml of streptomycin, 100 units/ml of penicillin, and 10% of heat-inactivated foetal bovine serum in humidified, 5% (v/v) CO_2_ atmosphere at 37 °C. Cytotoxic activity is expressed in terms of IC_50_ and is provided in (Table 1).

**Table 1.** *In vitro* cytotoxicy results against T-47D cell line.

Compound ID	IC_50_ (µM)
**IIIa**	5
**IIIj**	12.5
**Vib**	12

#### 3.2.3. *In vitro* initial screening of PI3K enzyme inhibitory activity for 3 isoforms at a single dose of 10 µm concentration

The *in vitro* PI3K kinase inhibition assay was performed at ThermoFisher Scientific (Madison, WI USA) using Adapta™ screening protocol.

The % inhibitory effect of the compounds on PI3K isoforms activities was assessed at 10 μM concentration against PI-103 as a reference compound. The observed activities of the compounds are mentioned in Table 2. Compound **VIb** bearing 3-hydroxyphenyl at 2nd position of thienopyrimidine scaffold demonstrated the best activity against both PI3Kβ and PI3Kγ isoforms with inhibitory activity 72% and 84%, respectively. On the other hand, compound **IIIa** showed good activity on both PI3Kβ and PI3Kγ isoforms with inhibitory activity of 62% and 70%, respectively.

**Table 2.** The inhibitory activity of synthesised compounds on PI3K isoforms. Green coded number is moderate activity (40%–80%). Red coded number is significant activity (above 80%).

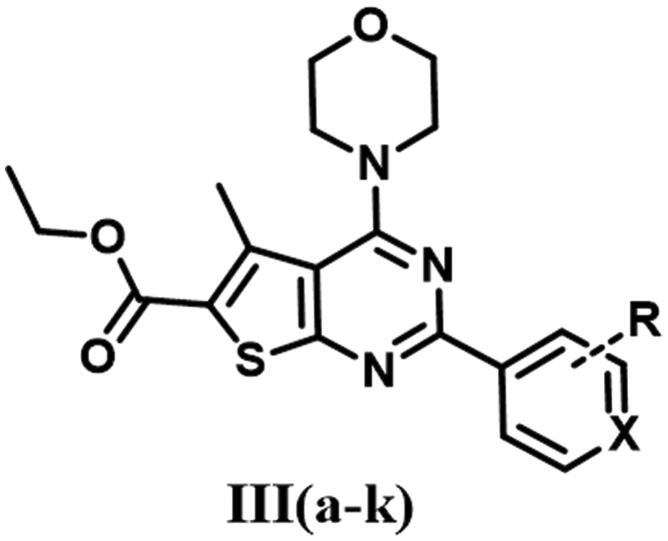		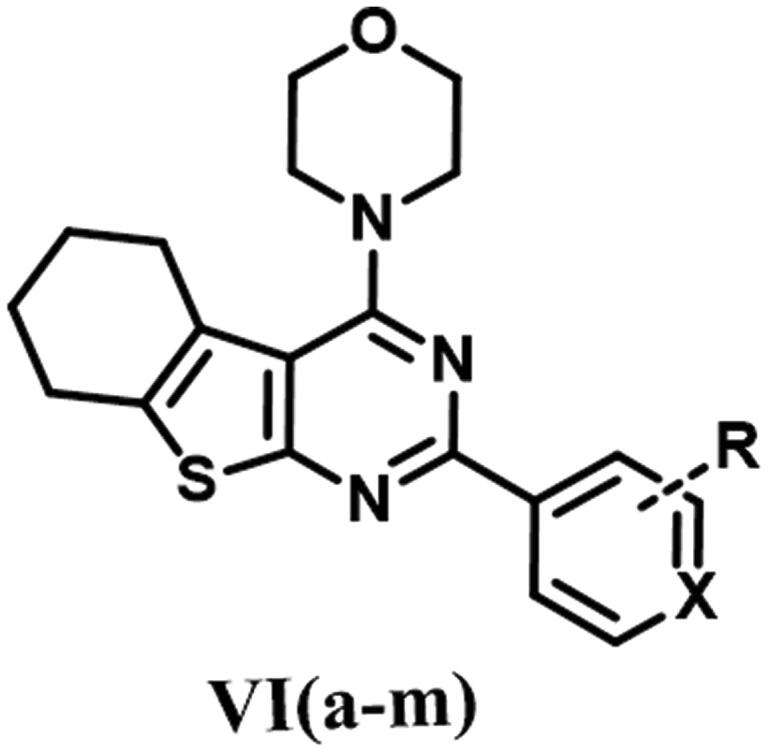	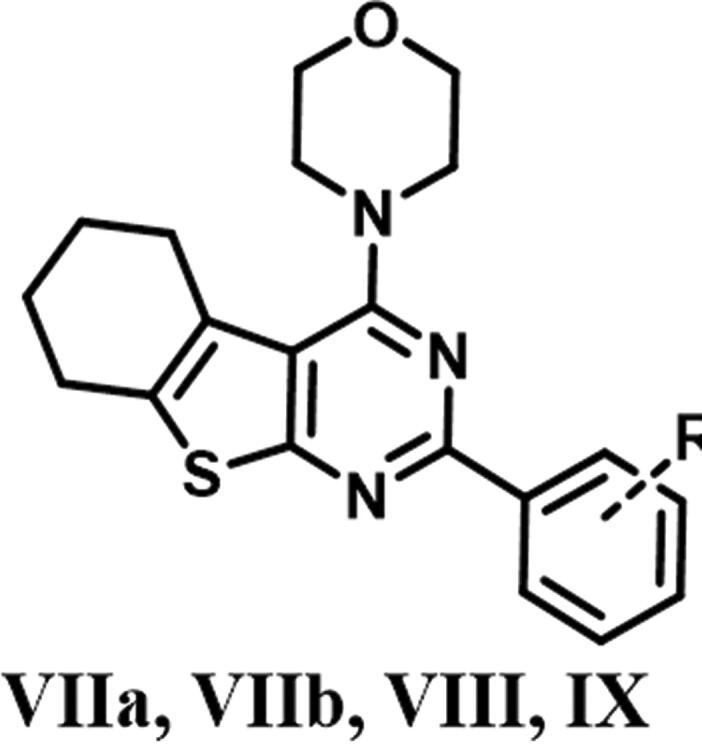	
Compound ID	R, X	PI3Kα % inhibition	PI3Kβ % inhibition at 10µM	PI3Kγ % inhibition
**IIIa**	R = 3-OH, X = CH	37	62	70
**IIIb**	R = 4-OH, X = CH	30	39	33
**IIIc**	R = 3-OCH_3_, X = CH	23	33	23
**IIId**	R = 4-OCH_3_, X = CH	25	22	17
**IIIe**	R = 4-Cl, X = CH	23	2	15
**IIIf**	R = 3-NO_2_, X = CH	11	9	7
**IIIg**	R = 4-NO_2_, X = CH	14	−12	11
**IIIh**	R = 3-CN, X = CH	16	12	15
**IIIi**	R = 4-(N(CH_3_)_2_), X = CH	11	25	22
**IIIj**	R = 3,4,5-Trimethoxy, X = CH	7	17	6
**IIIk**	R = H, X = N	19	28	48
**VIa**	R = H, X = CH	9	3	4
**VIb**	R = 3-OH, X = CH	27	72	84
**VIc**	R = 4-OH, X = CH	24	50	36
**VId**	R = 3-OCH_3_, X = CH	4	8	10
**VIe**	R = 4-OCH_3_, X = CH	15	17	1
**VIf**	R = 3-Cl, X = CH	0	−5	7
**VIg**	R = 4-Cl, X = CH	3	−6	12
**VIh**	R = 3-NO_2_, X = CH	5	−13	12
**VIi**	R = 4-NO_2_, X = CH	11	−15	6
**VIj**	R = 3-CN, X = CH	15	−12	13
**VIk**	R = 4-(N(CH_3_)_2_), X = CH	10	−7	2
**VIl**	R = 3,4,5-Trimethoxy, X = CH	15	−13	10
**VIm**	R = H, X = N	17	29	38
**VIIa**	R = 3-NH_2_	8	13	35
**VIIb**	R-4-NH_2_	15	1	16
**VIII**	R = 4-NHCOCH_3_	3	7	12
**IX**	R = 4-(4-Methylbenzamido)phenyl	15	11	8
**IC_50_** (**nM**)	PI-103 (performed at Thermofisher Scientific)	132	5.37	14

However, compound **VIc** showed moderate activity on PI3Kβ isoform as its inhibitory activity is 50%, compound **IIIk** showed moderate activity on PI3Kγ isoform as its inhibitory activity is 48%. Changing the position of hydroxyl from 3 to 4 decreased the inhibitory activity as in compounds **IIIb** and **VIc**. Unfortunately, replacing the OH group with bioisosteric NH_2_ resulted in a significant drop in activity, also acylation derivatives did not show any significant inhibitory activity.

### 3.3. Field alignment and molecular docking

#### 3.3.1. Field alignment

As a preliminary evaluation for our rationally designed compounds, a molecular field alignment study was performed to predict whether the designed compounds will have a comparable binding mode to the co-crystallized potent inhibitor PI-103 (4) inside the binding site of PI3Kα and good binding to the co-crystallized potent inhibitor GDC-0941 (1) inside the binding site of PI3Kβ and PI3Kγ. Molecules interact through their electronic properties (electrostatic and van der Waals forces), and proteins perceive the ligands as molecular fields rather than mere 3D arrangements of individual atoms. Therefore, molecules with similar potential fields (similar set of field points) are expected to exert similar biological activities through establishing the same interactions within the same binding site, even if they have very diverse structures, since field patterns are not directly affected by 2D connectivity of the molecule but rather by its 3D properties^58–59^.

Based on this theory, our design strategy involved the use of the *in-silico* field alignment technique provided by Cresset’s FieldAlign^®^ module, version 2.0.1 in an attempt to illustrate the similarity of the molecular fields between the designed compounds and PI-103 (4) (as reference inhibitor for PI3Kα) and GDC-0941 (1) (as reference inhibitor for PI3Kβ and PI3Kγ). The designed compounds were aligned to the bioactive conformation of PI-103 (4) (PDB: 4L23) and the bioactive conformation of GDC-0941 (1) (PDB: 2Y3A and PDB: 3DBS) to form the field alignment pattern which is represented as field points with different colour codes indicating negative, positive, van der Waals and hydrophobic fields. Larger field points represent stronger points of potential interaction. The designed compounds showed (55–75%) field alignment to bioactive conformation’s field pattern of the reference molecules.

**Figure 6.**
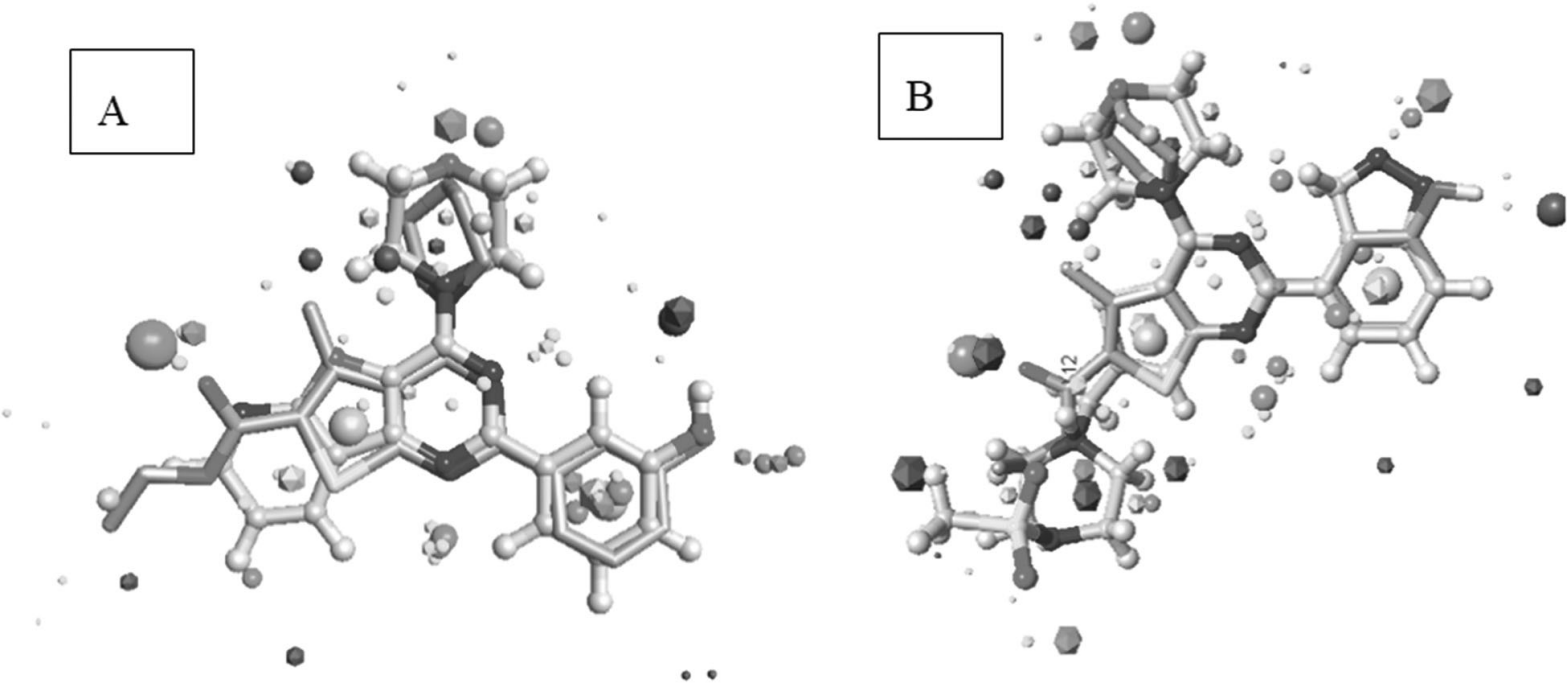
(A) Field alignment of compound IIIa and PI-103 showing similar molecular fields suggesting a similar binding mode to PI3Kα (with score 0.76). (B) Field alignment of compound **IIIa** and GDC-0941showing similar molecular fields suggesting a similar binding mode to PI3Kβ and PI3Kγ (with score 0.61). Spherical field points: compound **IIIa**, icosahedral field points: reference compound. Cyan: Negative field points, Red: Positive field points, Yellow: van der Waals surface field points, Gold: Hydrophobic field points, compound **IIIa** is displayed in grey.

**Figure 7.**
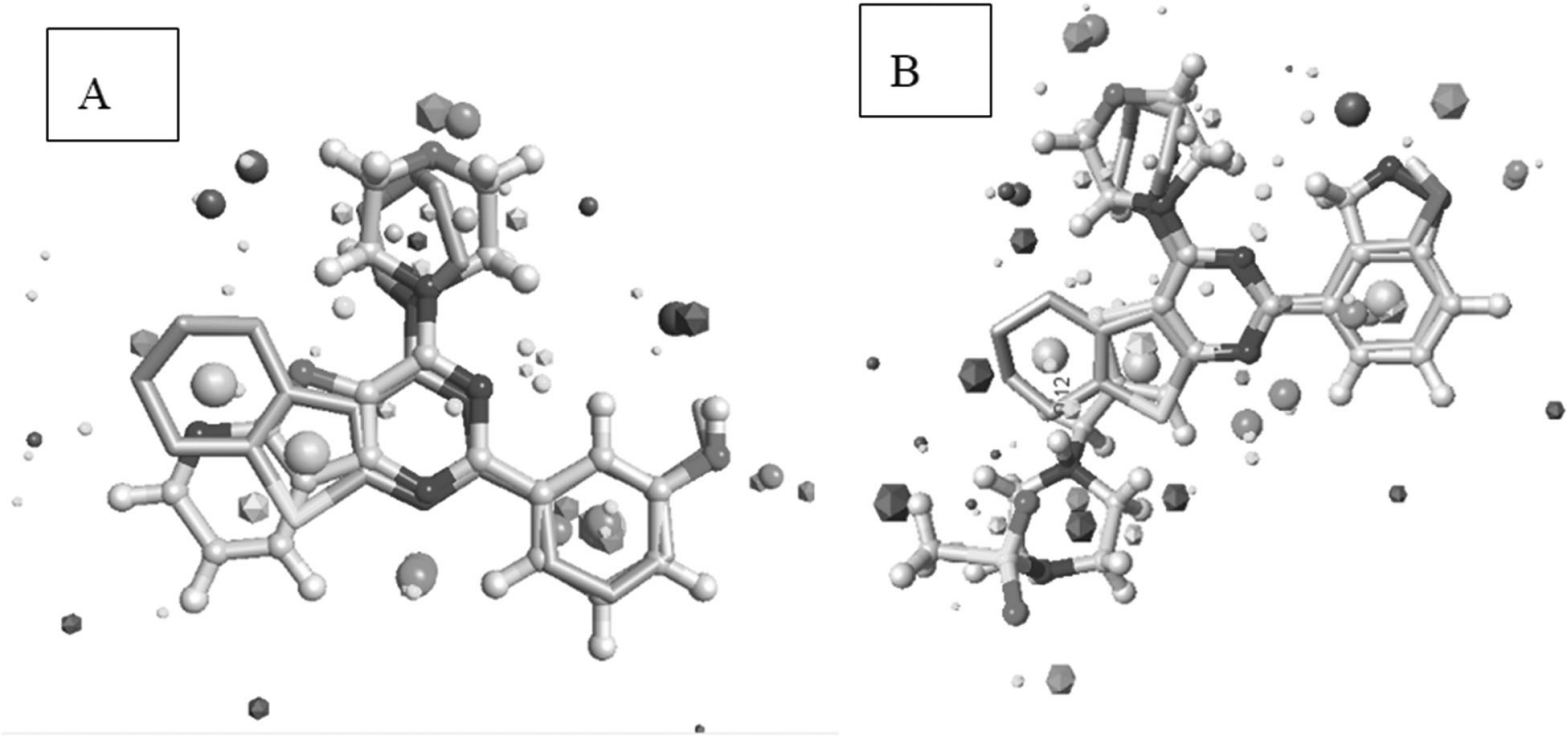
(A) Field alignment of compound **VIb** and PI-103 showing similar molecular fields suggesting a similar binding mode to PI3Kα (with score 0.786). (B) Field alignment of compound **VIb** and GDC-0941showing similar molecular fields suggesting a similar binding mode to PI3Kβ and PI3Kγ (with score 0.658). Spherical field points: compound **VIb**, icosahedral field points: reference compound. Cyan: Negative field points, Red: Positive field points, Yellow: van der Waals surface field points, Gold: Hydrophobic field points, compound **VIb** is displayed in grey. Colour Codes – Cyan: Negative field points, Red: Positive field points, Yellow: van der Waals surface field points, Gold: Hydrophobic field points

The designed compounds showed similar electronic fields as the lead compound with a field alignment score above (0.6) suggesting that they can form similar interactions with the protein as that of the lead (Figures 6 and 7). They have negative field points in the position that is proposed to bind with Val residue in the hinge region. They also showed a similar hydrophobic field with negative field points in the position that is proposed to bind with Tyr, Asp, and/or Lys. This was illustrated through the overlapping between field points representing the designed compounds and the field points representing reference compounds. Besides, the alignment agrees with our predicted binding mode from the docking study. It is worth noting that compounds **IIIa** and **VIb**, which had the highest inhibitory potency showed the best alignment score.

#### 3.3.2. Molecular docking

A molecular modelling study was carried out through docking of the target compounds in the binding site of different PI3K isoforms using C-DOCKER protocol in Accelrys Discovery Studio^®^ 2.5. Computational docking is an automated computer-based algorithm used to estimate the suitable pose (orientation & conformation) of the target compounds inside the binding site compared to that of the X-ray crystallographic enzyme-substrate complex as well as calculation of the docking scoring, which is the estimated protein-ligand interaction energy^60^.

Docking was carried out using C-DOCKER protocol for PI3Kα, β and PI3Kγ. C-DOCKER (CHARMm-based Docker) protocol used in this study is a grid-based MD docking algorithm, which offers all the advantages of full ligand flexibility (including bonds, angles, and dihedrals), the CHARMm family of force fields, the flexibility of the CHARMm engine, and reasonable computation times^61^. This docking study was performed to investigate the SAR of the target compounds, and as an attempt to interpret the biological evaluation results on the basis of the ligand-protein interactions.

The X-ray crystal structure of PI3Kα (PDB: 4L23) co-crystallized with the lead compound PI-103 (4) was used in this study. The X-ray crystal structure of compound GDC-0941 (1) co-crystallized with PI3Kβ (PDB: 2Y3A) and PI3Kγ (PDB: 3DBS) also are used in this study. The binding mode and the interactions with PI3K were discussed earlier.

**Figure 8.**
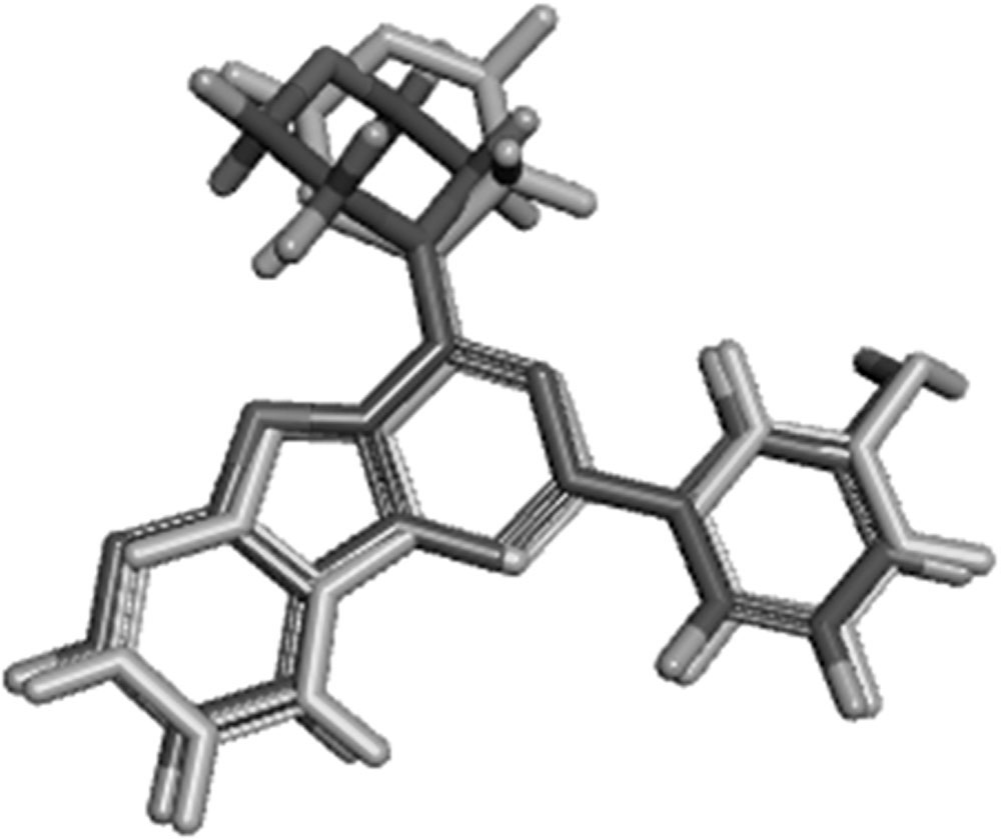
The alignment between the co-crystallized bioactive conformer of lead compound PI-103(4) [yellow colored] and its pose of retrieved from docking using C-DOCKER.

**Figure 9.**
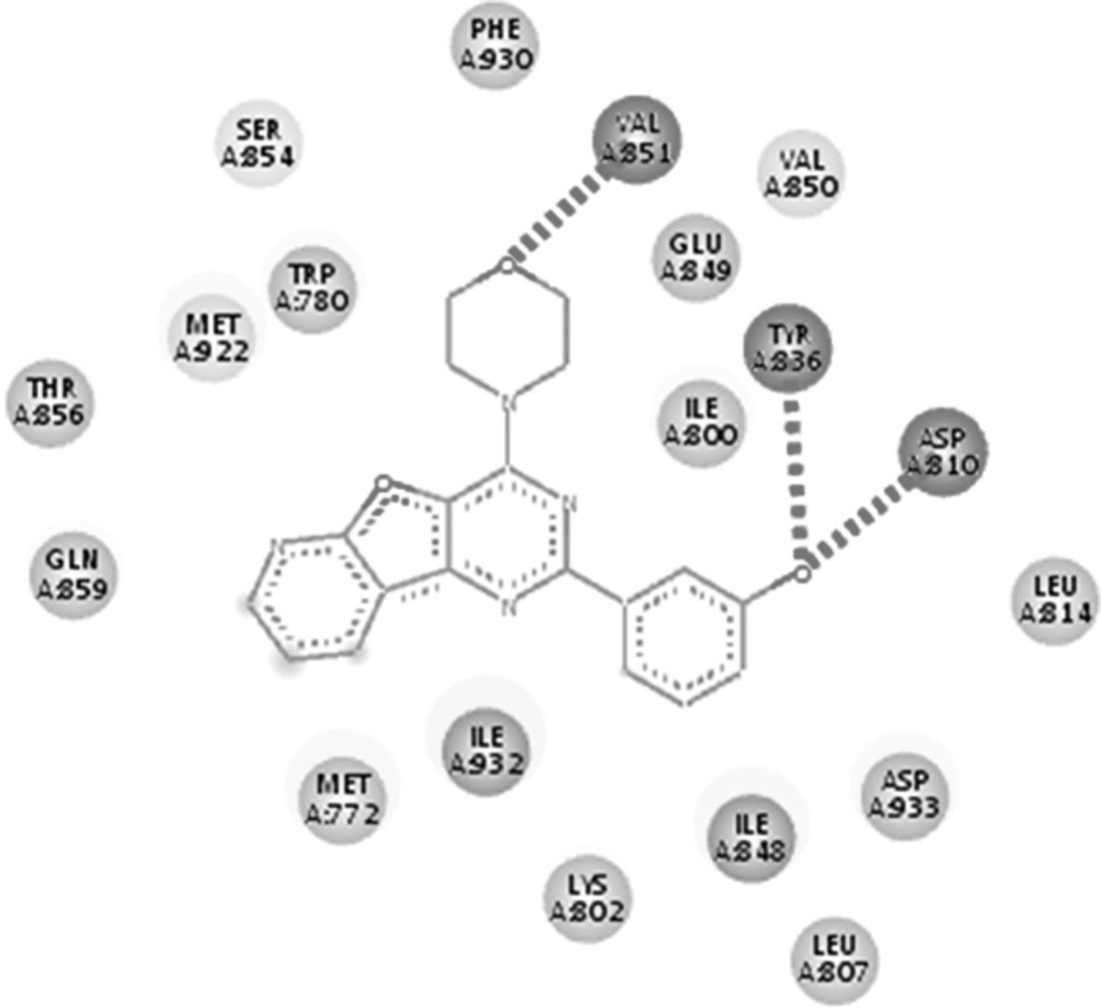
The 2D interaction of re-docked PI-103(4) with PI3Kα.

**Figure 10.**
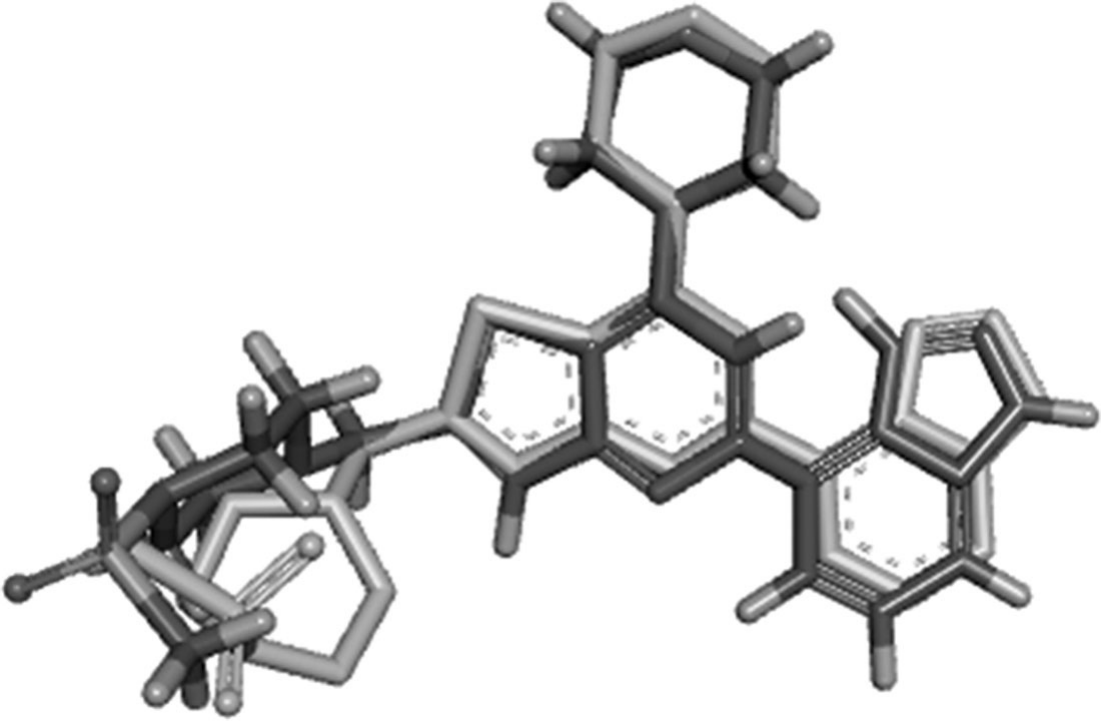
The alignment between the co-crystallized bioactive conformer of GDC-0941(1) [yellow colored] and its pose of retrieved from docking using C-DOCKER.

**Figure 11.**
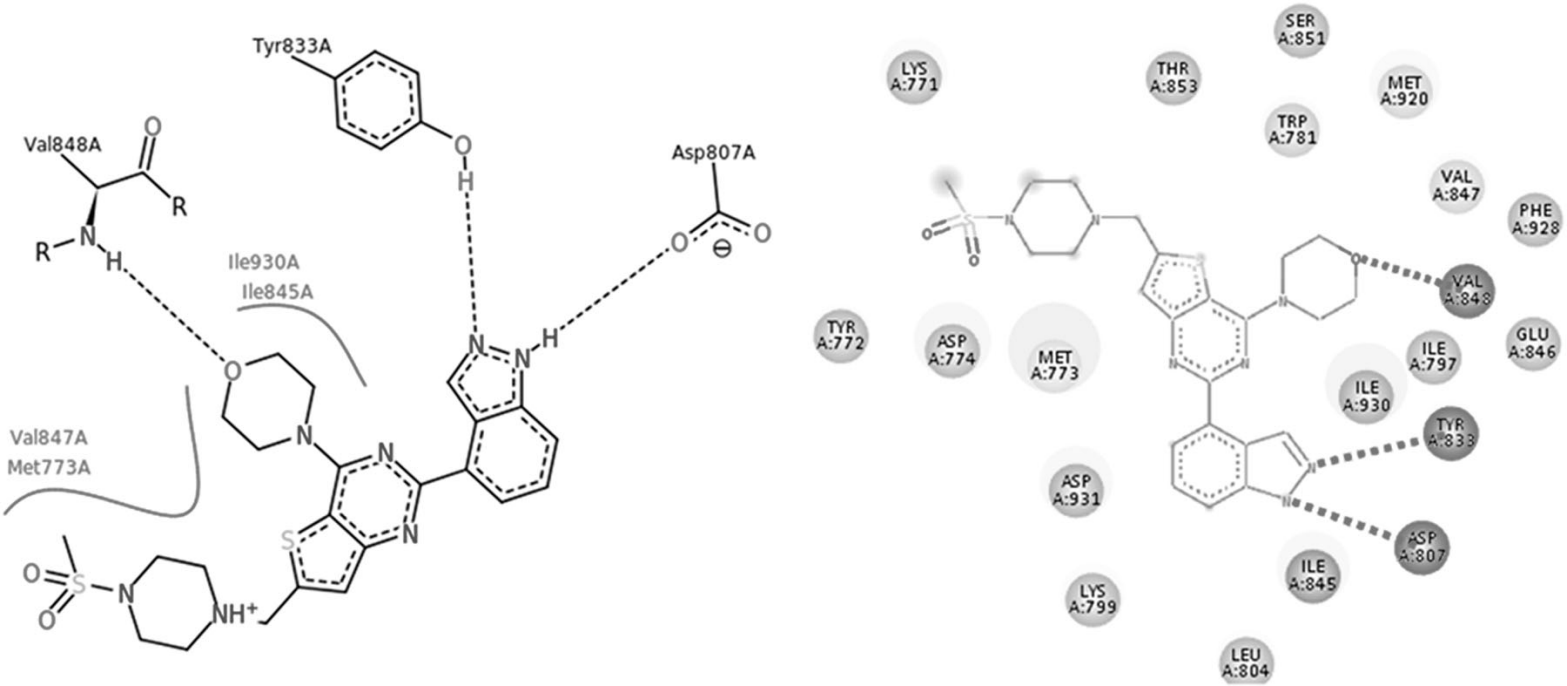
The reported 2D interaction of GDC-0941 (PDB: 2Y3A) vs. re-docked GDC-0941 with PI3Kβ.

**Figure 12.**
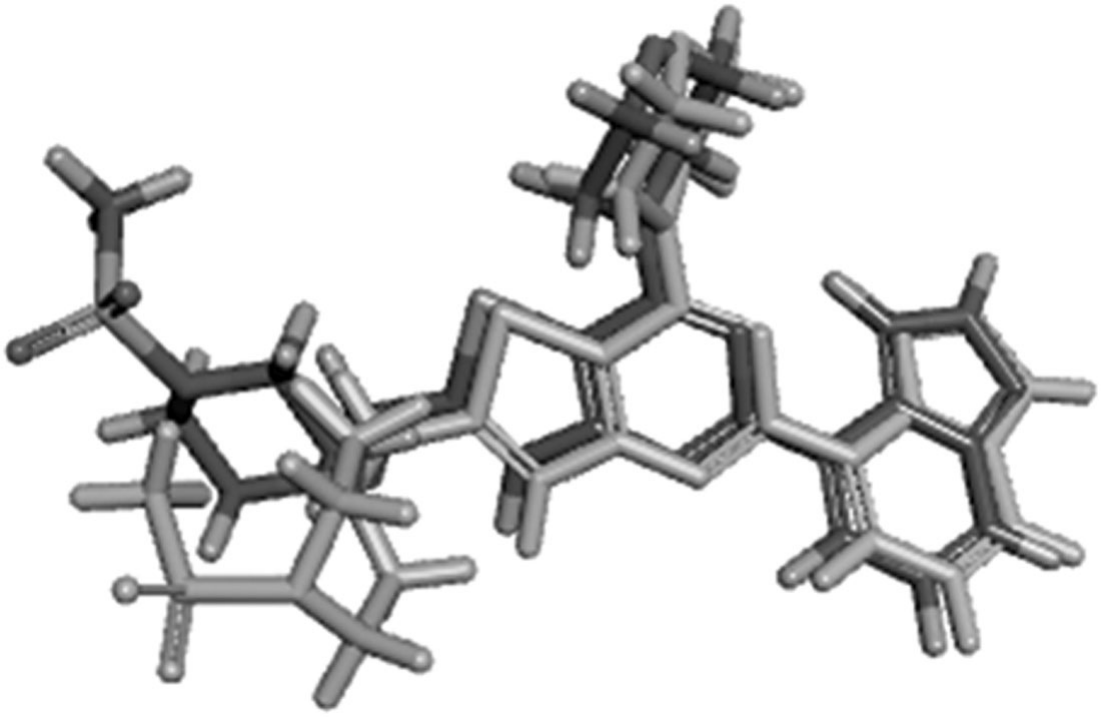
The alignment between the co-crystallized bioactive conformer of GDC-0941(1) [yellow colored] and its pose of retrieved from docking using C-DOCKER.

**Figure 13.**
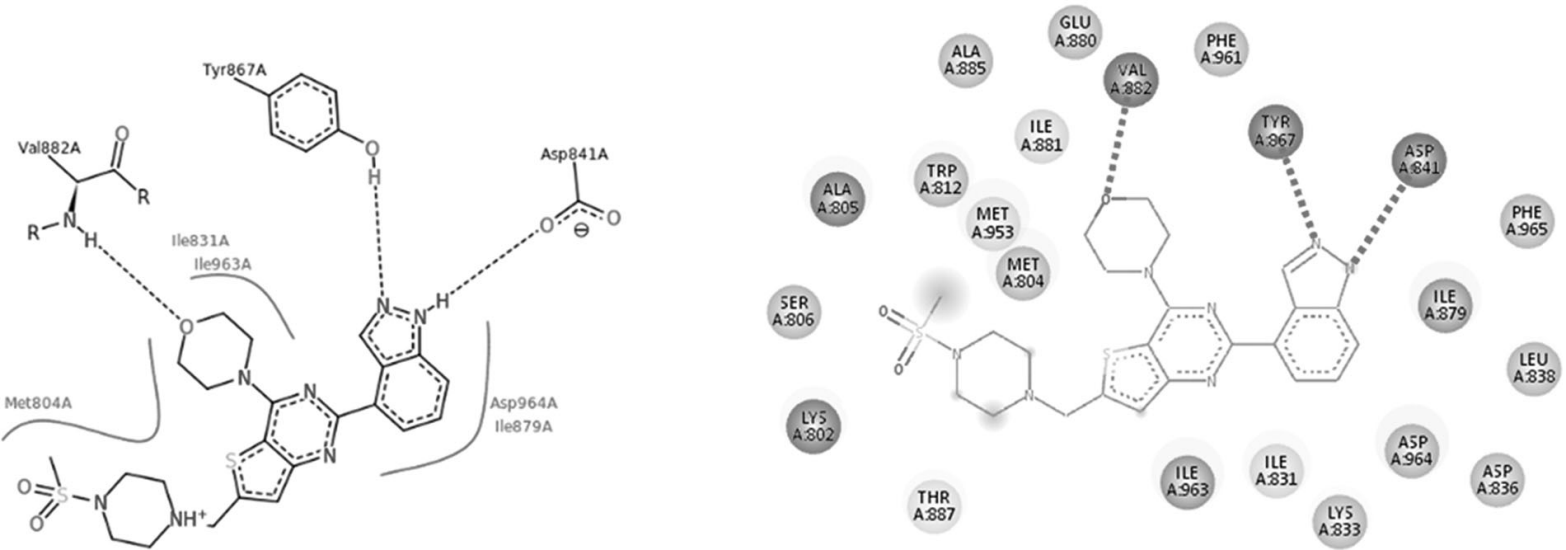
The reported 2D interaction of GDC-0941 (PDB: 3DBS) vs. re-docked GDC-0941 with PI3Kγ.

The docking protocol aims to predict the pose (orientation and conformation) of the ligand with respect to the binding site of the protein. Therefore, the major goal of a good docking protocol is to discriminate between proposed poses, usually defined as poses within 2.0 Å root mean square deviations (RMSD) from the X-ray geometry, and false or misdocked poses. In order to validate the C-DOCKER protocol’s predictability of the correct poses, we re-docked the co-crystallized ligand PI-103 (4) using C-DOCKER, and aligned the pose retrieved from docking to the X-ray geometry (the co-crystallized bioactive conformation) to calculate the RMSD. The docking of PI-103 (4) in PI3Kα (PDB: 4L23) generated an RMSD of 0.3267 Å (Figure 8) with the same interaction as reported (Figure 9). The docking of GDC-0941 (1) in PI3Kβ (PDB: 2Y3A) generated an RMSD of 0.4158 Å (Figure 10) with the same interaction as reported (Figure 11). The docking of GDC-0941 (1) in PI3Kγ (PDB: 3DBS) generated RMSD of 0.5236 Å (Figure 12) with the same interaction as reported (Figure 13), suggesting the validation of C-DOCKER protocol in predicting the pose of the inhibitors for all 3 isoforms (PI3Kα, PI3Kβ as well as. PI3Kγ).

**Figure 14.**
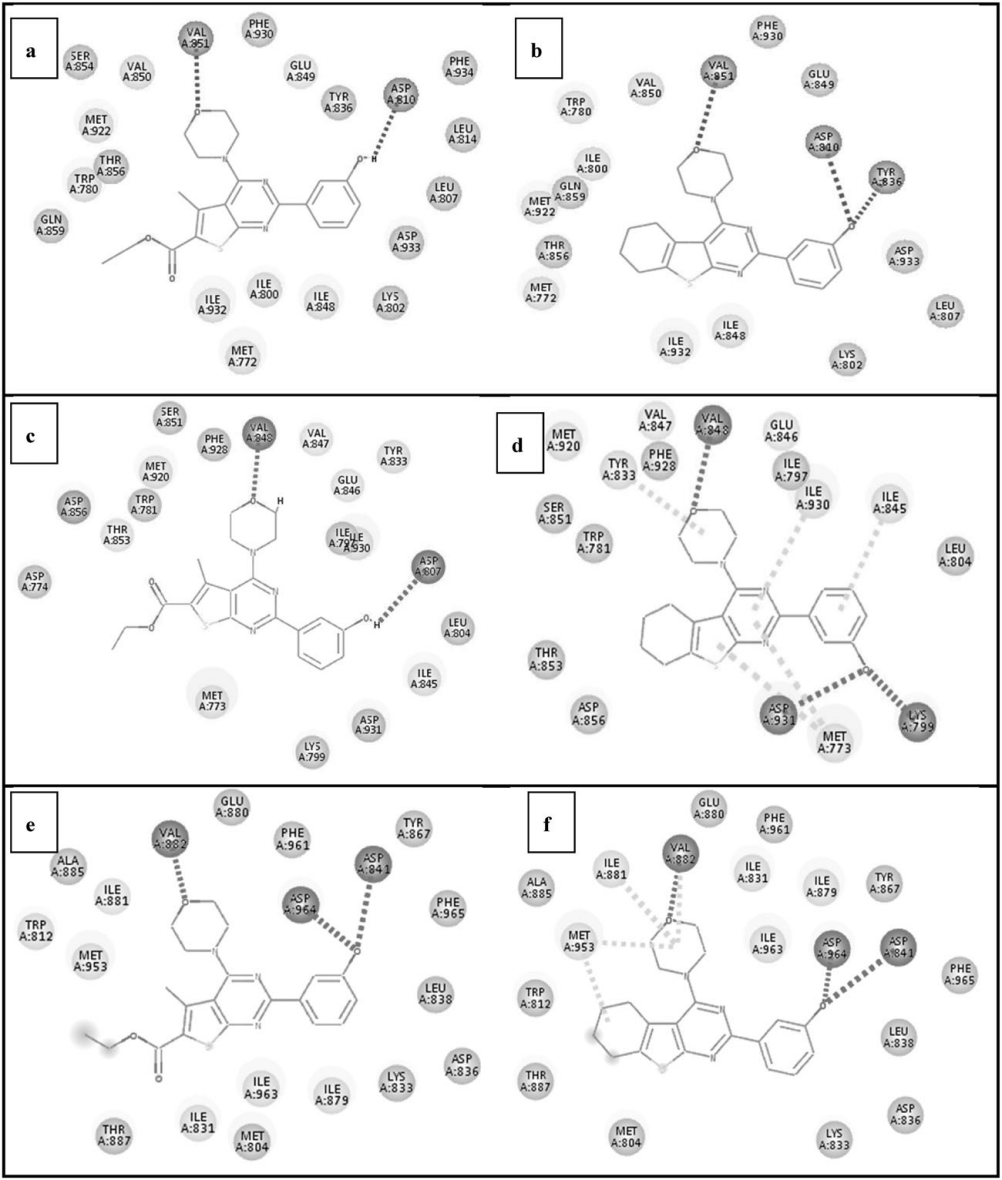
(a) Docking pose of compound (**IIIa**) showing the binding interactions against PI3Kα (4L23) (with score −40.2474). (b) Docking pose of compound (**VIb**) showing the binding interactions against PI3Kα (4L23) (with score −42.57). (c) Docking pose of compound (**IIIa**) showing the binding interactions against PI3Kβ (2Y3A) (with score −41.799). (d) Docking pose of compound (**VIb**) showing the binding interactions against PI3Kβ (2Y3A) (with score −43.8). (e) Docking pose of compound (**IIIa**) showing the binding interactions against PI3Kγ (3DBS) (with score −44.8). (f) Docking pose of compound (**VIb**) showing the binding interactions against PI3Kγ (3DBS) (with score −46.53).

The binding interactions of the docked compounds **IIIa** and **VIb** together with their binding energies are presented in Figure 14. The loss of one of key HB with either Tyr836, Asp810 or Lys802 residues may explain the poor enzymatic activity of **IIIa** against PI3Kα. Moreover, docking study may explain the good enzymatic activity of **IIIa** against PI3Kβ and γ. The binding of compound **IIIa** with PI3Kβ showed 2HBs between morpholine moiety and Val848 as well as 3-hydroxyl group and Asp807 giving C-DOCKER interaction energy of −41.799 Kcal/mol. Similar results were revealed upon docking **IIIa** with PI3Kγ as it formed main HB with Val882 and 2HBs with Tyr867 and Asp841 giving C-DOCKER interaction energy of −44.8 Kcal/mol.

The binding of compound **VIb** is relatively comparable to the lead compound (GDC-0941) (1). It fulfilled the basic HBs by its morpholine moiety with Val848 residue which resembles the key HB of compound (GDC-0941) (1). Additionally, 2HB interactions were established between its 3-hydroxyl moiety and Lys799 and Asp931 residues as well as additional hydrophobic interaction of thienopyrimidine ring with Met773 giving C-DOCKER interaction energy of −43.8 Kcal/mol on PI3Kβ. Similar results were revealed upon docking **VIb** with PI3Kγ as it formed 3 HBs with Val882, Asp964, and Asp841 with additional hydrophobic interaction between tetrahydrohexane and Met953. Another hydrophobic interaction was formed between morpholine moiety and Ile881 resulting in C-DOCKER interaction energy of −46.53 Kcal/mol on PI3Kγ.

The 50% enzymatic activity of **VIc** against PI3Kβ can be explained by docking study as it formed main HB with Val848 PI3Kβ and compensated the loss of 2HBs with Tyr833 and Asp807 by forming HB with Lys799 and additional hydrophobic interaction between thienopyrimidine core and Met773 in β isoform giving C-DOCKER interaction energy of −42.321 Kcal/mol on PI3Kβ.

Compounds with high docking scores maintain the main interactions with PI3K or compensate the loss of main interactions by forming additional hydrophobic interactions with surrounding amino acids, mainly Met, Ile, and Tyr in different PI3K isoforms.

## 4. Conclusion

Several novel approaches to target PI3K and its pathways have been identified in recent years through synthesising small molecule inhibitors. Based on literature review and SAR studies, novel series of 2-aryl-4-morpholinothieno[2,3-*d]* pyrimidine derivatives were designed, synthesised, and evaluated for their *in vitro* PI3K inhibitory activity against different isotypes PI3Kα, β and γ as well as their anti-proliferative activity against NCI 60 cell lines. Compounds (**IIIa** and **VIb**), having 3-OH substitution on the phenyl ring, showed good enzymatic activity on PI3Kβ (62% and 72%), respectively and on PI3Kγ (70% and 84%), respectively as well as good inhibitory activity against most of the NCI cell lines with mean growth inhibition percent of (41.8% and 47.3%), respectively. Moreover, derivatives having more lipophilic tetramethylene substitution at 5&6 positions of thienopyrimidine core generally showed better activity compared to their corresponding 5-methyl-6-carboxylate analogues (**VIb, c** vs. **IIIa, b**). Compound (**VIb**) exhibited the best enzymatic inhibitory activity (72% & 84% on PI3Kβ & PI3Kγ), respectively, and good activity on most NCI cell lines especially those with over-expressed PI3K.

Hence, based on this study, the crucial features to be considered for the design of an effective PI3K inhibitor can be summarised in keeping the morpholine moiety to maintain PI3K inhibitory activity as it binds to Val residue in the hinge region. After screening various substitutions on phenyl ring attached to 2nd position of thienopyrimidine scaffold, the best activity resulted from 3-OH moiety. Transferring 3-OH to para position results in a significant decrease in the inhibitory activity. Changing OH by other groups abolished the enzymatic activity even if replaced by its bioisosteric NH_2_. The extension strategy did not result in any improvement of the activity.

## 5. Experimental section

### 5.1. Chemistry

Starting material, reagents and solvents were purchased from Sigma-Aldrich (Germany), Alfa-Aesar Organics (Germany), Merck (Darmstadt, Germany), and Loba Chemie (India) and utilised without further purification processes. Solvents used for column chromatography were redistilled before use on BUCHI Rotavapor. Flash column chromatography was performed using silica gel (230–400 mesh particle size) purchased from Sigma-Aldrich. Reactions were monitored utilising TLC plates which are silica gel 60 F254 stuffed on Aluminium sheets and envisioned under U.V light (254 nm) and the plates were purchased from Merck. Ethyl acetate/Hexane (3:7) mixture was used as an eluting solvent. Melting points were measured in capillary tubes on Stuart Scientific apparatus and reported without correction. FT-IR spectra were performed using a Thermo Scientific Nicolet iS10 spectrometer at Ain Shams University. ^1^H-NMR and ^13^C NMR spectra were recorded in the *δ* scale given in ppm on Bruker 400 MHz and 100 MHz spectrophotometer, respectively and alluded to TMS as an internal indicator at the Centre for Drug Discovery Research and Development, faculty of pharmacy, Ain Shams University. Both electron impact mass spectra (EI-MS) and elemental analyses were recorded by Thermo Scientific ISQLT mass spectrometer and Thermo Scientific Flash 2000 elemental analyser respectively, at the Regional Centre for Mycology and Biotechnology, Al-Azhar University. All yields were calculated before further purification.

#### 5.1.1. General procedure for preparation of compounds I and IV

The targeted compounds I^34^ and IV^35^ were prepared through the “Gewald Aminothiophene Synthesis”^29^ which is a one-pot reaction that involves a multi-component condensation between an α-methylene carbonyl compounds and activated nitriles with elemental sulphur in presence of a base to produce 2-aminothiophene derivatives. In the present study, the titled 2-aminothiophene (**I**, **IV**) were obtained by reacting cyanoacetamide (38.5 mmol, 1 equivalent) with ethyl acetoacetate or cyclohexanone (38.5 mmol, 1 equivalent) and elemental sulphur (38.5 mmol, 1 equivalent) in polar solvent DMF containing morpholine as base^30–35^. The product was assessed for its formation using TLC. The reaction mixture was heated overnight at 50–60 °C until complete dissolving of sulphur occurred. The reaction mixture was then poured drop wise on ice (100 ml) to give precipitate. The precipitate was filtered and washed with excess water to get rid of excess DMF and left to dry to give the titled compounds.

##### 5.1.2. General procedure for preparation of intermediate compounds IIa–k and Va–e and Vg–m

To a solution of compounds (**I** or **IV**) (3.29 mmol, 1 equivalent) in dry DMF, the appropriate benzaldehyde derivative (3equivalents) was added. Conc HCl (0.2 ml) was added to this reaction mixture and heated overnight at 110 °C or until TLC shows the completion of reaction^36–38^. After completion of the reaction, the reaction was poured into ice-cold water with vigorous stirring where precipitate was formed. The precipitate was filtered and washed with hexane to yield target compounds **IIa–k** and **Va–e**, and **Vg–m)**. The intermediates formation was assessed by using TLC and m.p for reported compounds (**Va**^62^, **Vd**^35^, **Ve**^35^, **Vg**^35^, **Vh**^35^, **Vi**^62^, **Vl**^35^)

##### 5.1.3. General procedure for preparation of intermediate compound (Vf)

A suspension of the 3-chlorobenzoic acid (12.77 mmol, 1 equivalent) in dry DCM (15 ml) was cooled to 0 °C in ice bath. Thionyl chloride (63.87 mmol, 5 equivalents) was added dropwise with stirring, then the reaction mixture was stirred under reflux for 9 h^63^. The solvent was then evaporated under vacuum giving a brownish solid of the respective acid chloride that was used directly without further purification. To a stirred solution of the titled compound (**IV**) (3.82 mmol, 1 equivalent) in dry DCM (10 ml) at 0 °C, a solution of 3-chlorobenzoyl chloride (5.348 mmol, 1.4 equivalent) and 4 drops of TEA in dry DCM (10 ml) were added dropwise^63^. Then the ice bath was removed and the reaction was stirred at room temperature for 24 h. Then, the reaction mixture was poured to ice/H_2_O/10% HCl (15 ml) with vigorous stirring where precipitate was formed.

To a suspension of 2-(3-chlorobenzamido)-4,5,6,7-tetrahydrobenzo[*b*]thiophene-3-carboxamide (**A**)^40^ (1.28 g, 3.82 mmol, 1equivalent) in 10 ml absolute isopropanol, 2 N sodium hydroxide (10 ml) was added and then the reaction mixture was refluxed for 12 h^41–42^. After cooling to room temperature, the reaction mixture was concentrated to remove isopropanol then poured into iced water (30 ml). The solution was then acidified by the addition of hydrochloric acid (5 ml), at 0 °C. The titled compound (**Vf**)^39^ was formed and collected by filtration then washed with hexane.

#### 5.1.4. General procedure for preparation 4-chlorothieno[2,3-d]pyrimidine derivatives

A mixture of the appropriate respective thieno[2,3-*d*] pyrimidin-4-one derivatives (**IIa–k**) and (**Va–m**) (3.14 mmol, 1 equivalent) and POCl_3_ (59.36 mmol, 18.9 equivalent) was cooled to 0 °C in an ice bath during the addition of POCl_3_. POCl_3_ was added dropwise with stirring reaction mixture. Then the mixture was heated under reflux for 4–12 h^43–44^. The mixture was then slowly poured on ice/water, then neutralised using ammonia solution (33%, 20 ml), then extracted with ethyl acetate (2*50 ml). The combined organic layer was separated, dried over anhydrous Na_2_SO_4_ and the solvent was evaporated under vacuum to afford the chlorinated compounds (40–80%). The formation of 4-chloro derivatives was monitored by TLC. They were directly involved in the next reaction without purification due to their high reactivity and moisture sensitivity.

#### 5.1.5. General procedure for preparation targeted compounds (IIIa–k) and (VIa–m)

To a solution of the 4-chlorothieno[2,3-*d*]pyrimidine derivatives (2.13 mmol, 1 equivalent) in a mixture of ethanol: isopropanol (1:1) (15 ml), morpholine (2.13 mmol, 1 equivalent) and 4 drops of TEA were added dropwise^45^. The reaction mixture was heated at 80 °C. The formation of targeted derivatives was monitored by TLC. The precipitate was filtered, washed with an appropriate solvent, allowed to dry and then the titled compounds were purified by using the flash column chromatography to afford the target compounds (**IIIa–k**) and (**VIa–m**).

##### 5.1.5.1. Ethyl 2-(3-hydroxyphenyl)-5-methyl-4-morpholinothieno[2,3-d]pyrimidine-6-carboxylate (IIIa)

The titled compound (**IIIa**) was purified using the flash column chromatography (gradient elution hexane: ethyl acetate = 9:1 till 7:3) and separated as pale orange powder (0.83 g, 85.26%); m.p. 218–220 °C; ^1^H-NMR (400 MHz, DMSO-d_6_): *δ* (ppm) 9.61 (s, 1H, OH), 7.93–7.72 (m, 2H, ArH), 7.30 (s, 1H, ArH), 6.92 (d, *J* = 7.0 Hz, 1H, ArH), 4.32 (q, *J* = 7.0 Hz, 2H, OCH_2__)_, 3.78 (t, *J = 5.5 Hz,* 4H, morpholine), 3.53 (t, *J = 4.8 Hz*, 4H, morpholine), 2.74 (s, 3H, CH_3_), 1.32 (t, *J* = 7.1 Hz, 3H, OCH_2_CH_3_). ^13^C NMR (100 MHz, DMSO-d_6_): *δ* (ppm) 169.53, 163.42, 162.54, 159.64, 158.01, 139.79, 138.50, 129.97, 122.80, 119.50, 118.67, 118.49, 115.23, 66.21, 61.67, 50.86, 16.85, 14.61. MS (Mwt =399.47), *m/z* (% rel. int.): 399.36 (M^+^, 100%), 400.32 (M^+^+H, 60.41%). Anal. Calcd. for C_20_H_21_N_3_O_4_S: C, 60.14; H, 5.30; N, 10.52; S, 8.03; Found: C, 59.97; H, 5.46; N, 10.79; S, 8.11. FT-IR (*ύ* max, cm^−1^): 3393.6 (phenolic OH stretch), 3005 (CH aromatic), 2964.42 (CH aliphatic), 1707.64 (C = O ester).

##### 5.1.5.2. Ethyl 2-(4-hydroxyphenyl)-5-methyl-4-morpholinothieno[2,3-d]pyrimidine-6-carboxylate (IIIb)

The titled compound (**IIIb**) was purified using the flash column chromatography (gradient elution hexane: ethyl acetate = 9:1 till 1:1) and separated as off white powder (0.8 g, 87.32%); m.p. 230–232 °C; ^1^H-NMR (400 MHz, DMSO-d_6_): *δ* (ppm) 10.04 (s, 1H, OH), 8.27 (d, *J* = 8.0 Hz, 2H, ArH), 6.89 (d, *J* = 8.2 Hz, 2H, ArH), 4.33 (q, *J = 7.1 Hz*, 2H, OCH_2_), 3.78 (t, *J = 5.8 Hz*, 4H, morpholine), 3.53 (t, *J = 5.5 Hz*, 4H, morpholine), 2.77 (s, 3H, CH_3_), 1.33 (t, *J = 7.0 Hz*, 3H, OCH_2_CH_3_). MS (Mwt =399.47), *m/z* (% rel. int.): 399.36 (M^+^, 100%), 400.32 (M^+^+H, 21.42%). Anal. Calcd. for C_20_H_21_N_3_O_4_S: C, 60.14; H, 5.30; N, 10.52; S, 8.03; Found: C, 60.28; H, 5.43; N, 10.75; S, 8.13. FT-IR (*ύ* max, cm^−1^): 3448.24 (phenolic OH stretch), 3010 (CH aromatic), 2962.09 (CH aliphatic), 1676.83 (C = O ester).

##### 5.1.5.3. Ethyl 2-(3-methoxyphenyl)-5-methyl-4-morpholinothieno[2,3-d]pyrimidine-6-carboxylate (IIIc)

The titled compound (**IIIc**) was purified using the flash column chromatography (gradient elution DCM: hexane = 9:1 then changed to DCM and finally DCM: ethyl acetate 9:1) and separated as dark beige powder (0.95 g, 93.66%); m.p. 117–119 °C; ^1^H-NMR (400 MHz, CDCl_3_-d_1_): *δ* (ppm) 8.17–8.01 (m, 2H, ArH), 7.41 (t, *J = 7.9* Hz, 1H, ArH), 7.13–7.01 (m, 1H, ArH), 4.41 (q, *J = 7.1 Hz*, 2H, OCH_2_), 3.93 (d, *J = 5.5 Hz*, 7H, morpholine & OCH_3_), 3.62 (t, *J = 4.7 Hz*, 4H, morpholine), 2.86 (s, 3H, CH_3_), 1.44 (t, *J = 7.1 Hz*, 3H, OCH_2_CH_3_). MS (Mwt =413.49), *m/z* (% rel. int.): 413.21 (M^+^, 100%), 414.30 (M^+^+H, 42.83%). Anal. Calcd. for C_21_H_23_N_3_O_4_S: C, 61; H, 5.61; N, 10.16; S, 7.75; Found: C, 60.79; H, 5.88; N, 10.40; S, 7.69. FT-IR (*ύ* max, cm^−1^): 3013 (CH aromatic), 2968.78 (CH aliphatic), 1708.21 (C = O ester).

##### 5.1.5.4. Ethyl 2-(4-methoxyphenyl)-5-methyl-4-morpholinothieno[2,3-d]pyrimidine-6-carboxylate (IIId)

The titled compound (**IIId**) was purified using the flash column chromatography (gradient elution DCM: hexane = 9:1 then changed to DCM and finally DCM: ethyl acetate 9:1) and separated as light beige powder (0.96 g, 93.6%); m.p. 154–156 °C; ^1^H-NMR (400 MHz, DMSO-d_6_): *δ* (ppm) 8.34 (d, *J = 7.7 Hz*, 2H, ArH), 7.05 (d, *J = 7.5 Hz*, 2H, ArH), 4.32 (q, *J = 7.1 Hz*, 2H, OCH_2_), 3.84 (s, 3H, OCH_3_), 3.78 (t, *J = 4.9 Hz*, 4H, morpholine), 3.51 (t, *J = 5.6 Hz*, 4H, morpholine), 2.74 (s, 3H, CH_3_), 1.33 (t, *J = 7.1 Hz*, 3H, OCH_2_CH_3_). ^13^C NMR (100 MHz, DMSO-d_6_): *δ* (ppm) 169.74, 163.48, 162.63, 162.17, 159.57, 139.93, 130.34, 127.82, 122.22, 118.26, 114.42, 66.23, 61.64, 55.81, 50.87, 16.88, 14.65. MS (Mwt =413.49), *m/z* (% rel. int.): 413.23 (M^+^, 100%), 414.30 (M^+^+H, 39.35%). Anal. Calcd. for C_21_H_23_N_3_O_4_S: C, 61; H, 5.61; N, 10.16; S, 7.75; Found: C, 61.17; H, 5.84; N, 10.38; S, 7.79. FT-IR (*ύ* max, cm^−1^): 3010 (CH aromatic), 2930.28 (CH aliphatic), 1700.78 (C = O ester).

##### 5.1.5.5. Ethyl 2-(4-chlorophenyl)-5-methyl-4-morpholinothieno[2,3-d]pyrimidine-6-carboxylate (IIIe)

The titled compound (**IIIe**) was purified using the flash column chromatography (gradient elution DCM: hexane = 9:1 then changed to DCM and finally DCM: ethyl acetate 9:1) and separated as off white powder (0.98 g, 87.88%); m.p. 146–148 °C; ^1^H-NMR (400 MHz, CDCl_3_-d_1_): *δ* (ppm) 8.44 (d, *J* = 8.3 Hz, 2H, ArH), 7.46 (d, *J* = 8.0 Hz, 2H, ArH), 4.41 (q, *J = 7.1 Hz*, 2H, OCH_2_), 3.93 (t, *J = 4.7 Hz*, 4H, morpholine), 3.61 (t, *J = 4.6 Hz*, 4H, morpholine), 2.86 (s, 3H, CH_3_), 1.44 (t, *J = 7.1 Hz*, 3H, OCH_2_CH_3_). MS (Mwt = 417.91), *m/z* (% rel. int.): 417.14 (M^+^, 100%), 418.18 (M^+^+H, 33.05%), 419.15 (M ^+ 2^,41.92%). Anal. Calcd. for C_20_H_20_ClN_3_O_3_S: C, 57.48; H, 4.82; N, 10.06; S, 7.67; Found: C, 57.71; H, 4.98; N, 9.89; S, 7.80. FT-IR (*ύ* max, cm^−1^): 3003 (CH aromatic), 2956.97 (CH aliphatic), 1701.56 (C = O ester).

##### 5.1.5.6. Ethyl 5-methyl-4-morpholino-2-(3-nitrophenyl)thieno[2,3-d]pyrimidine-6-carboxylate (IIIf)

The titled compound (**IIIf**) was purified using the flash column chromatography (gradient elution DCM: hexane = 9:1 then changed to DCM and finally DCM: ethyl acetate 9.5:0.5) and separated as light beige powder (0.88 g, 87.18%); m.p. 216–218 °C; ^1^H-NMR (400 MHz, DMSO-d_6_): *δ* (ppm) 9.08 (s, 1H, ArH), 8.76 (d, *J* = 8.3 Hz, 1H, ArH), 8.36 (d, *J* = 8.2 Hz, 1H, ArH), 7.81 (t, *J* = 8.0 Hz, 1H, ArH), 4.34 (q, *J* = 7.1 Hz, 2H, OCH_2_), 3.80 (t, *J = 4.5 Hz*, 4H, morpholine), 3.59 (t, *J = 4.9 Hz*, 4H, morpholine), 2.76 (s, 3H, CH_3_), 1.34 (t, *J* = 7.1 Hz, 3H, OCH_2_CH_3_). MS (Mwt = 428.46), *m/z* (% rel. int.): 428.26 (M^+^, 100%), 429.35 (M^+^+H, 36.7%). Anal. Calcd. for C_20_H_20_N_4_O_5_S: C, 56.07; H, 4.71; N, 13.08; S, 7.48; Found: C, 56.29; H, 4.87; N, 13.34; S, 7.57. FT-IR (*ύ* max, cm^−1^): 3092.83 (CH aromatic), 2966.98 (CH aliphatic), 1708.34 (C = O ester), 1496.72–1363.90 (NO_2_ stretch).

##### 5.1.5.7. Ethyl 5-methyl-4-morpholino-2-(4-nitrophenyl)thieno[2,3-d]pyrimidine-6-carboxylate (IIIg)

The titled compound (**IIIg**) was purified using the flash column chromatography (gradient elution hexane: ethyl acetate = 9:1 till 7:3) and separated as yellow powder (0.9 g, 85.33%); m.p. 189–191 °C; ^1^H-NMR (400 MHz, DMSO-d_6_): *δ* (ppm) 8.61 (d, *J* = 8.5 Hz, 2H, ArH), 8.35 (d, *J* = 8.6 Hz, 2H, ArH), 4.35 (q, *J* = 7.1 Hz, 2H, OCH_2_), 3.80 (t, *J* = 5.3 Hz, 4H, 4H, morpholine), 3.62 (t, *J* = 5.2 Hz, 4H, morpholine), 2.77 (s, 3H, CH_3_), 1.34 (t, *J* = 7.0 Hz, 3H, OCH_2_CH_3_). MS (Mwt = 428.46), *m/z* (% rel. int.): 428.19 (M^+^, 100%), 429.27 (M^+^+H, 35.36%). Anal. Calcd. for C_20_H_20_N_4_O_5_S: C, 56.07; H, 4.71; N, 13.08; S, 7.48; Found: C, 56.31; H, 4.84; N, 13.30; S, 7.60. FT-IR (*ύ* max, cm^−1^): 3008 (CH aromatic), 2926.96 (CH aliphatic), 1701.40 (C = O ester), 1497.38–1345.25 (NO_2_ stretch).

##### 5.1.5.8. Ethyl 2-(3-cyanophenyl)-5-methyl-4-morpholino-thieno[2,3-d]pyrimidine-6-carboxylate (IIIh)

The titled compound (**IIIh**) was purified using the flash column chromatography (gradient elution hexane: ethyl acetate = 9:1 till 1:1) and separated as off white powder (0.98 g, 90.36%); m.p. 176–178 °C; ^1^H-NMR (400 MHz, DMSO-d_6_): *δ* (ppm) 8.62 (s, 2H, ArH), 7.97 (d, *J* = 7.7 Hz, 1H, ArH), 7.71 (t, *J* = 8.0 Hz, 1H, ArH), 4.32 (q, *J* = 7.2 Hz, 2H, OCH_2_), 3.78 (t, *J* = 4.7 Hz, 4H, morpholine), 3.56 (t, *J* = 4.9 Hz, 4H, morpholine), 2.73 (s, 3H, CH_3_), 1.34 (t, *J* = 7.4 Hz, 3H, OCH_2_CH_3_). ^13^C NMR (100 MHz, DMSO- d_6_): *δ* (ppm) 169.33, 163.33, 162.42, 157.45, 139.70, 138.25, 134.66, 132.87, 131.68, 130.43, 123.55, 119.04, 118.98, 112.31, 66.23, 61.78, 50.83, 16.96, 14.61. MS (Mwt = 408.48), *m/z* (% rel. int.): 408.33 (M^+^, 100%), 409.32 (M^+^+H, 32.27%). Anal. Calcd. for C_21_H_20_N_4_O_3_S: C, 61.75; H, 4.94; N, 13.72; S, 7.85; Found: C, 61.94; H, 5.12; N, 14.01; S, 8.02. FT-IR (*ύ* max, cm^−1^): 3203.15 (CH aromatic), 2980.71 (CH aliphatic), 2204.27 (C≡N), 1701.61 (C = O ester).

##### 5.1.5.9. Ethyl 2-(3-(dimethylamino) phenyl)-5-methyl-4-morpholinothieno[2,3-d] pyrimidine-6-carboxylate (IIIi)

The titled compound (**IIIi**) was purified using the flash column chromatography (gradient elution hexane: ethyl acetate = 9:1 till 7:3) and separated as dark yellow powder (0.93 g, 91.06%); m.p. 118–120 °C; ^1^H-NMR (400 MHz, CDCl_3_-d_1_): *δ* (ppm) 8.4 (dd, *J* = 8.3, 2.5 Hz, 2H, ArH), 6.79 (dd, *J* = 8.2, 2.4 Hz, 2H, ArH), 4.35 (q, *J* = 7.2 Hz, 2H, OCH_2_), 3.83 (t, *J = 4.7 Hz*, 4H, morpholine), 3.59 (t, *J = 4.9 Hz*, 4H, morpholine), 3.08 (s, 6H, N(CH_3_)_2_), 2.54 (s, 3H, CH_3_), 1.39 (t, *J* = 7.4 Hz, 3H, OCH_2_CH_3_). MS (Mwt = 426.54), *m/z* (% rel. int.): 426.29 (M^+^, 100%), 427.35 (M^+^+H, 31.42%). Anal. Calcd. for C_22_H_26_N_4_O_3_S: C, 61.95; H, 6.14; N, 13.14; S, 7.52; Found: C, 62.13; H, 6.29; N, 13.38; S, 7.48.

##### 5.1.5.10. Ethyl 5-methyl-4-morpholino-2-(3,4,5-trimethoxyphenyl)thieno[2,3-d] pyrimidine-6-carboxylate (IIIj)

The titled compound (**IIIj**) was purified using the flash column chromatography (gradient elution hexane: ethyl acetate = 9:1 till 1:1) and separated as light brown powder (0.97 g, 88.39%); m.p. 152–154 °C; ^1^H-NMR (400 MHz, DMSO-d_6_): *δ* (ppm) 7.72 (s, 2H, ArH), 4.34 (q, *J* = 7.1 Hz, 2H, OCH_2_), 3.89 (s, 6H, 3,5(OCH_3_)_2_), 3.80 (d, *J* = 5.3 Hz, 4H, morpholine), 3.76 (s, 3H, 4-methoxy), 3.57 (d, *J* = 5.4 Hz, 4H, morpholine), 2.77 (s, 3H, CH_3_), 1.34 (t, *J* = 7.1 Hz, 3H, OCH_2_CH_3_). ^13^C NMR (100 MHz, DMSO-d_6_): *δ* (ppm) 169.52, 163.34, 162.56, 159.16, 153.31, 140.65, 139.76, 132.44, 122.78, 118.55, 105.80, 66.21, 61.68, 60.60, 56.32, 50.86, 16.85, 14.61. MS (Mwt = 473.54), *m/z* (% rel. int.): 473.54 (M^+^, 100%), 474.33 (M^+^+H, 31.36%). Anal. Calcd. for C_23_H_27_N_3_O_6_S: C, 58.34; H, 5.75; N, 8.87; S, 6.77; Found: C, 58.60; H, 5.88; N, 9.09; S, 6.81. FT-IR (*ύ* max, cm^−1^): 3003.15 (CH aromatic), 2950.62 (CH aliphatic), 1709.65 (C = O ester).

##### 5.1.5.11. Ethyl 5-methyl-4-morpholino-2-(pyridin-4-yl)thieno[2,3-d]pyrimidine-6-carboxylate (IIIk)

The titled compound (**IIIk**) was purified using the flash column chromatography (gradient elution hexane: ethyl acetate = 7:3 till 3:7) and separated as pale yellow powder (0.94 g, 96.01%); m.p. 174–176 °C; ^1^H-NMR (400 MHz, DMSO-d_6_): *δ* (ppm) 8.76 (d, *J* = 7.4 Hz, 2H, ArH), 8.27 (d, *J* = 7.6 Hz, 2H, ArH), 4.35 (q, *J = 7.1 Hz*, 2H, OCH_2_), 3.78 (d, *J = 5.9* Hz, 4H, morpholine), 3.63 (d, *J = 5.3* Hz, 4H, morpholine), 2.78 (s, 3H, CH_3_), 1.34 (t, *J = 7.1 Hz*, 3H, OCH_2_CH_3_). MS (Mwt = 384.45), *m/z* (% rel. int.): 384.45 (M^+^, 100%). Anal. Calcd. for C_19_H_20_N_4_O_3_S: C, 59.36; H, 5.24; N, 14.57; S, 8.34; Found: C, 59.62; H, 5.38; N, 14.79; S, 8.45.

##### 5.1.5.12. 4-Morpholino-2-phenyl-5,6,7,8-tetrahydrobenzo[4,5]thieno[2,3-d]pyrimidine (VIa)

The titled compound (**VIa**) was purified using the flash column chromatography (gradient elution DCM: hexane = 9:1 till DCM) and separated as white powder (0.98 g, 83.87%), m.p. 156–158 °C; ^1^H-NMR (400 MHz, CDCl_3_-d_1_): *δ* (ppm) 8.57–8.41 (m, 2H, ArH), 7.57–7.41 (m, 3H, ArH), 4.00–3.85 (m, 4H, morpholine), 3.53 (m, 4H, morpholine), 2.94 (t, *J = 4.8 Hz*, 4H, cyclohexyl CH_2_), 2.03–1.79 (m, 4H, cyclohexyl CH_2_). ^13^C NMR (100 MHz, DMSO-d_6_): *δ* (ppm) 168.95, 162.12, 157.05, 137.76, 135.02, 130.69, 128.98, 128.08, 127.83, 119.41, 66.32, 51.13, 26.60, 25.80, 23.01, 22.73. MS (Mwt = 351.47), *m/z* (% rel. int.): 351.27 (M^+^, 100%), 352.28 (M^+^+H, 22.65%). Anal. Calcd. for C_20_H_21_N_3_OS: C, 68.35; H, 6.02; N, 11.96; S, 9.12; Found: C, 68.58; H, 6.19; N, 12.24; S, 8.97. FT-IR (*ύ* max, cm^−1^): 3040.05 (CH aromatic), 2955.2 (CH aliphatic).

##### 5.1.5.13. 2-(3-Hydroxyphenyl)-4-morpholino-5,6,7,8-tetrahydrobenzo[4,5]thieno[2,3-d]-pyrimidine (VIb)

The titled compound (**VIb**) was purified using the flash column chromatography (gradient elution hexane: ethyl acetate = 9:1 till 7:3) and separated as light beige powder (0.88 g, 79.86%), m.p. 199–201 °C; ^1^H-NMR (400 MHz, CDCl_3_-d_1_): *δ* (ppm) 8.06 (d, *J* = 7.8 Hz, 1H, ArH), 7.97 (s, 1H, ArH), 7.36 (t, *J* = 7.9 Hz, 1H, ArH), 6.96 (d, *J* = 7.2 Hz, 1H, ArH), 3.94 (t, *J* = 5.1 Hz 4H, morpholine), 3.53 (t, *J* = 4.9 Hz, 4H, morpholine), 2.93 (m, 4H, cyclohexyl CH_2_), 1.99–1.80 (m, 4H, cyclohexyl CH_2_). MS (Mwt = 367.47), *m/z* (% rel. int.): 367.30 (M^+^, 100%), 368.34 (M^+^+H, 30.14%). Anal. Calcd. for C_20_H_21_N_3_O_2_S: C, 65.37; H, 5.76; N, 11.44; S, 8.72; Found: C, 65.49; H, 5.89; N, 11.71; S, 8.61. FT-IR (*ύ* max, cm^−1^): 3448.98 (phenolic OH), 2918.77 (CH aliphatic).

##### 5.1.5.14. 2-(4-Hydroxyphenyl)-4-morpholino-5,6,7,8-tetrahydrobenzo[4,5]thieno[2,3-d]-pyrimidine (VIc)

The titled compound (**VIc**) was purified using the flash column chromatography (gradient elution DCM: hexane = 9:1 till DCM) and separated as off white powder (1 g, 86.21%), m.p. 210–212 °C; ^1^H-NMR (400 MHz, CDCl_3_-d_1_): *δ* (ppm) 8.37 (d, *J* = 8.2 Hz, 2H, ArH), 6.93 (d, *J* = 8.3 Hz, 2H, ArH), 6.11 (s, 1H, OH), 3.98–3.89 (m, 4H, morpholine), 3.53 (t, *J* = 4.7 Hz, 4H, morpholine), 2.92 (m, 4H, cyclohexyl CH_2_), 1.98–1.93 (m, 2H, cyclohexyl CH_2_), 1.85 (d, *J* = 6.7 Hz, 2H, cyclohexyl CH_2_). ^13^C NMR (100 MHz, Chloroform-d_1_): *δ* (ppm) 161.90, 158.28, 134.87, 130.01, 126.89, 119.03, 115.50, 66.70, 51.01, 26.75, 25.83, 23.04, 22.88. MS (Mwt = 367.47), *m/z* (% rel. int.): 367.24 (M^+^, 100%), 368.32 (M^+^+H, 30.64%). Anal. Calcd. for C_20_H_21_N_3_O_2_S: C, 65.37; H, 5.76; N, 11.44; S, 8.72; Found: C, 65.58; H, 5.68; N, 11.70; S, 8.76. FT-IR (*ύ* max, cm^−1^): 3321.87 (phenolic OH), 2968.47 (CH aliphatic).

##### 5.1.5.15. 2-(3-Methoxyphenyl)-4-morpholino-5,6,7,8-tetrahydrobenzo[4,5]thieno[2,3-d]-pyrimidine (VId)

The titled compound (**VId**) was purified using the flash column chromatography (gradient elution DCM: hexane = 9:1 till DCM) and separated as white powder (0.99 g, 86.72%), m.p. 116–118 °C; ^1^H-NMR (400 MHz, CDCl_3_-d_1_): *δ* (ppm) 8.10 (d, *J* = 7.8 Hz, 1H, ArH), 8.08 (s, 1H, ArH), 7.40 (t, *J* = 7.9 Hz, 1H, ArH), 7.02 (t, *J* = 7.8 Hz 1H, ArH), 4.01 (s, 3H, OCH_3_), 3.94–3.92 (m, 4H, morpholine), 3.62–3.45 (m, 4H, morpholine), 2.94 (m, 4H, cyclohexyl CH_2_), 1.97 (m, 2H, cyclohexyl CH_2_), 1.85 (m, 2H, cyclohexyl CH_2_). MS (Mwt = 381.49), *m/z* (% rel. int.): 381.27 (M^+^, 100%), 382.28 (M^+^+H, 27.02%). Anal. Calcd. for C_21_H_23_N_3_O_2_S: C, 66.12; H, 6.08; N, 11.01; S, 8.40; Found: C, 66.40; H, 6.35; N, 11.28; S, 8.32. FT-IR (*ύ* max, cm^−1^): 3002.15 (CH aromatic), 2997.57 (CH aliphatic).

##### 5.1.5.16. 2-(4-Methoxyphenyl)-4-morpholino-5,6,7,8-tetrahydrobenzo[4,5]thieno[2,3-d]-pyrimidine (VIe)

The titled compound (**VIe**) was purified using the flash column chromatography (gradient elution hexane: ethyl acetate = 9:1 till 1:1) and separated as light beige powder (1.1 g, 90.85%), m.p. 146–148 °C; ^1^H-NMR (400 MHz, CDCl_3_-d_1_): *δ* (ppm) 8.47 (d, *J* = 8.2 Hz, 2H, ArH), 7.01 (d, *J* = 8.2 Hz, 2H, ArH), 3.92 (d, *J* = 4.5 Hz, 4H, morpholine), 3.90 (s, 3H, OCH_3_), 3.55 (m, 4H, morpholine), 3.09–2.71 (m, 4H, cyclohexyl CH_2_), 1.96 (q, *J* = 5.7 Hz, 2H, cyclohexyl CH_2_), 1.85 (s, 2H, cyclohexyl CH_2_). MS (Mwt = 381.49), *m/z* (% rel. int.): 381.32 (M^+^, 100%), 382.19 (M^+^+H, 31.98%). Anal. Calcd. for C_21_H_23_N_3_O_2_S: C, 66.12; H, 6.08; N, 11.01; S, 8.40; Found: C, 66.35; H, 6.92; N, 11.25; S, 8.47. FT-IR (*ύ* max, cm^−1^): 3015 (CH aromatic), 2934.66 (CH aliphatic).

##### 5.1.5.17. 2-(3-Chlorophenyl)-4-morpholino-5,6,7,8-tetrahydrobenzo[4,5]thieno[2,3-d]-pyrimidine (VIf)

The titled compound (**VIf**) was purified using the flash column chromatography (gradient elution DCM: hexane = 1:1 till 3:1) and separated as white powder (0.95 g, 91.7%), m.p. 198–200 °C; ^1^H-NMR (400 MHz, DMSO-d_6_): *δ* (ppm) 8.33 (m, 2H, ArH), 7.63–7.44 (m, 2H, ArH), 3.80 (t, *J* = 4.4 Hz, 4H, morphine), 3.44 (t, *J* = 4.5 Hz, 4H, morphine), 2.90 (m, 4H, cyclohexyl CH_2_), 1.90 (p, *J* = 5.7 Hz, 2H, cyclohexyl CH_2_), 1.76 (q, *J* = 5.7 Hz, 2H, cyclohexyl CH_2_). MS (Mwt = 385.91), *m/z* (% rel. int.): 385.27 (M^+^, 100%), 386.29 (M^+^+H, 30.67%), 387.29 (M^+^+2, 32.38%). Anal. Calcd. for C_20_H_20_ClN_3_OS: C, 62.25; H, 5.22; N, 10.89; S, 8.31; Found: C, 62.43; H, 5.41; N, 11.05; S, 8.23. FT-IR (*ύ* max, cm^−1^): 3020 (CH aromatic), 2937.90 (CH aliphatic).

##### 5.1.5.18. 2-(4-Chlorophenyl)-4-morpholino-5,6,7,8-tetrahydrobenzo[4,5]thieno[2,3-d]-pyrimidine (VIg)

The titled compound (**VIg**) was purified using the flash column chromatography (gradient elution DCM: hexane = 1:1 till 3:1) and separated as white powder (1.16 g, 93.31%), m.p. 205–207 °C; ^1^H-NMR (400 MHz, DMSO-d_6_): *δ* (ppm) 8.38 (d, *J* = 7.8 Hz, 2H, ArH), 7.56 (d, *J* = 7.9 Hz, 2H, ArH), 3.80 (t, *J* = 4.6 Hz, 4H, morpholine), 3.44 (t, *J* = 4.4 Hz, 4H, morpholine), 2.89 (m, 4H, cyclohexyl CH_2_), 1.89 (m, 2H, cyclohexyl CH_2_), 1.76 (q, *J* = 5.6 Hz, 2H, cyclohexyl CH_2_). MS (Mwt = 385.91), *m/z* (% rel. int.): 385.27 (M^+^,100%), 386.31 (M^+^+H, 26.45%), 387.21 (M^+^+2, 38.39%). Anal. Calcd. for C_20_H_20_ClN_3_OS: C, 62.25; H, 5.22; N, 10.89; S, 8.31; Found: C, 62.13; H, 5.49; N, 11.16; S, 8.37. FT-IR (*ύ* max, cm^−1^): 3003 (CH aromatic), 2933.91 (CH aliphatic).

##### 5.1.5.19. 4-Morpholino-2-(3-nitrophenyl)-5,6,7,8-tetrahydrobenzo[4,5]thieno[2,3-d]-pyrimidine (VIh)

The titled compound (**VIh**) was purified using the flash column chromatography (gradient elution DCM: hexane = 9:1 till DCM) and separated as pale yellow powder (1.19 g, 94.36%), m.p. 218–220 °C; ^1^H-NMR (400 MHz, CDCl_3_-d_1_): *δ* (ppm) 9.31 (s, 1H, ArH), 8.87 (d, *J* = 7.7 Hz, 1H, ArH), 8.44–8.25 (m, 1H, ArH), 7.66 (t, *J* = 7.9 Hz, 1H, ArH), 4.11–3.87 (m, 4H, morpholine), 3.75–3.41 (m, 4H, morpholine), 2.96 (q, *J* = 6.0 Hz, 4H, cyclohexyl CH_2_), 1.99 (s, 2H, cyclohexyl CH_2_), 1.87 (s, 2H, cyclohexyl CH_2_). MS (Mwt = 396.47), *m/z* (% rel. int.): 396.31 (M^+^,100%), 397.36 (M^+^+H, 31.04%). Anal. Calcd. for C_20_H_20_N_4_O_3_S: C, 60.59; H, 5.08; N, 14.13; S, 8.09; Found: C, 60.41; H, 5.24; N, 14.34; S, 8.20.

##### 5.1.5.20. 4-Morpholino-2-(4-nitrophenyl)-5,6,7,8-tetrahydrobenzo[4,5]thieno[2,3-d]-pyrimidine (VIi)

The titled compound (**VIi**) was purified using the flash column chromatography (gradient elution DCM: hexane = 1:1 till DCM) and separated as dark yellow powder (1.25 g, 94.81%), m.p. 228–230 °C; ^1^H-NMR (400 MHz, DMSO-d_6_): *δ* (ppm) 8.60 (d, *J* = 8.3 Hz, 2H, ArH), 8.33 (d, *J* = 8.6 Hz, 2H, ArH), 3.90–3.75 (m, 4H, morpholine), 3.48 (t, *J* = 4.6 Hz, 4H, morpholine), 3.01–2.84 (m, 4H, cyclohexyl CH_2_), 1.90 (s, 2H, cyclohexyl CH_2_), 1.77 (s, 2H, cyclohexyl CH_2_). MS (Mwt = 396.47), *m/z* (% rel. int.): 396.17 (M^+^, 100%), 397.24 (M^+^+H, 33.42%). Anal. Calcd. for C_20_H_20_N_4_O_3_S: C, 60.59; H, 5.08; N, 14.13; S, 8.09; Found: C, 60.63; H, 5.32; N, 14.29; S, 8.24. FT-IR (*ύ* max, cm^−1^): 3008 (CH aromatic), 2927.37 (CH aliphatic), 1556.18–1340.45 (NO_2_ stretch).

##### 5.1.5.21. 2-(3-Cyanophenyl)-4-morpholino-5,6,7,8-tetrahydrobenzo[4,5]thieno[2,3-d]-pyrimidine (VIj)

The titled compound (**VIj**) was purified using the flash column chromatography (gradient elution DCM: hexane = 9:1 till DCM) and separated as off white powder (0.93 g, 91.46%), m.p. 186–188 °C; ^1^H-NMR (400 MHz, CDCl_3_-d_1_): *δ* (ppm) 8.81 (d, *J* = 8.2 Hz, 1H, ArH), 8.76 (s, 1H, ArH), 7.74 (d, *J* = 7.5 Hz, 1H, ArH), 7.61 (t, *J* = 7.8 Hz, 1H, ArH), 3.95–3.89 (m, 4H, morpholine), 3.64–3.57 (m, 4H, morpholine), 2.93 (t, *J* = 6.0 Hz, 4H, cyclohexyl CH_2_), 2.14–1.93 (m, 2H, cyclohexyl CH_2_), 1.93–1.73 (m, 2H, cyclohexyl CH_2_). MS (Mwt = 376.48), *m/z* (% rel. int.): 376.21 (M^+^, 100%), 377.25 (M^+^+H, 38.75%). Anal. Calcd. for C_21_H_20_N_4_OS: C, 67.00; H, 5.35; N, 14.88; S, 8.52; Found: C, 66.89; H, 5.47; N, 15.13; S, 8.61. FT-IR (*ύ* max, cm^−1^): 3005 (CH aromatic), 2933.64 (CH aliphatic), 2227.81 (C≡N stretch).

##### 5.1.5.22. 2-(4-Dimethyaminophenyl)-4-morpholino-5,6,7,8-tetrahydrobenzo[4,5]thieno-[2,3-d]pyrimidine (VIk)

The titled compound (**VIk**) was purified using the flash column chromatography (gradient elution hexane: ethyl acetate = 9:1 till 7:3) and separated as dark beige powder (0.95 g, 97.41%), m.p. 195–197 °C; ^1^H-NMR (400 MHz, DMSO-d_6_): *δ* (ppm) 8.22 (d, *J* = 8.6 Hz, 2H, ArH), 6.78 (d, *J* = 8.6 Hz, 2H, ArH), 3.80 (t, *J* = 4.5 Hz, 4H, morpholine), 3.60–3.50 (m, 4H, morpholine), 2.99 (s, 6H, N(CH_3_)_2_), 2.87 (m, 4H, cyclohexyl CH_2_), 1.88 (m, 2H, cyclohexyl CH_2_), 1.75 (m, 2H, cyclohexyl CH_2_). ^13^C NMR (100 MHz, DMSO-d_6_): *δ* (ppm) 169.25, 162.1, 158.18, 152.15, 139.38, 129.57, 127.75, 125.1, 118.32, 111.90, 66.36, 51.17, 26.56, 25.73, 23.05, 22.7. MS (Mwt = 394.54), *m/z* (% rel. int.): 394.31 (M^+^, 100%), 395.33 (M^+^+H, 28.10%). Anal. Calcd. for C_22_H_26_N_4_OS: C, 66.98; H, 6.64; N, 14.20; S, 8.13; Found: C, 66.75; H, 6.73; N, 14.46; S, 8.30. FT-IR (*ύ* max, cm^−1^): 3009 (CH aromatic), 2945.18 (CH aliphatic).

##### 5.1.5.23. 4-Morpholino-2-(3,4,5-trimethoxyphenyl)-5,6,7,8-tetrahydrobenzo[4,5]thieno-[2,3-d]pyrimidine (VIl)

The titled compound (**VIl**) was purified using the flash column chromatography (gradient elution hexane: ethyl acetate = 9:1 till 7:3) and separated as yellow powder (0.85 g, 94.06%), m.p. 198–200 °C; ^1^H-NMR (400 MHz, CDCl_3_-d_1_): *δ* (ppm) 7.81 (s, 2H, ArH), 4.02 (s, 6H, 3,5-(OCH_3_)_2_), 3.94 (d, *J* = 3.6 Hz, 7H, 4- OCH_3_ and morpholine), 3.52 (m, 4H, morpholine), 2.99–2.87 (m, 4H, cyclohexyl CH_2_), 2.02–1.94 (s, 2H, cyclohexyl CH_2_), 1.86 (s, 2H, cyclohexyl CH_2_). MS (Mwt = 441.55), *m/z* (% rel. int.): 441.35 (M^+^, 65.53%), 442.26 (M^+^+H, 17.56%). Anal. Calcd. for C_23_H_27_N_3_O_4_S: C, 62.56; H, 6.16; N, 9.52; S, 7.26; Found: C, 62.78; H, 6.30; N, 9.79; S, 7.31. FT-IR (*ύ* max, cm^−1^): 3002 (CH aromatic), 2932.51 (CH aliphatic).

##### 5.1.5.24. 4-Morpholino-2-(pyridine-4-yl)-5,6,7,8-tetrahydrobenzo[4,5]thieno[2,3-d]-pyrimidine (VIm)

The titled compound (**VIm**) was purified using the flash column chromatography (gradient elution hexane: ethyl acetate = 4:1 till 1:1) and separated as off white powder (0.9 g, 96.33%), m.p. 193–194 °C; ^1^H-NMR (400 MHz, DMSO-d_6_): *δ* (ppm) 8.73 (d, *J* = 5.1 Hz, 2H, ArH), 8.25 (d, *J* = 5.1 Hz, 2H, ArH), 3.87–3.74 (m, 4H, morpholine), 3.49 (m, 4H, morpholine), 2.91 (m, 4H, cyclohexyl CH_2_), 1.90 (s, 2H, cyclohexyl CH_2_), 1.76 (d, *J* = 7.7 Hz, 2H, cyclohexyl CH_2_). MS (Mwt = 352.46), *m/z* (% rel. int.): 352.33 (M^+^, 100%), 353.25 (M^+^+H, 29.26%). Anal. Calcd. for C_19_H_20_N_4_OS: C, 64.75; H, 5.72; N, 15.90; S, 9.10; Found: C, 65.02; H, 5.86; N, 16.13; S, 8.97. FT-IR (*ύ* max, cm^−1^): 3007 (CH aromatic), 2938.77 (CH aliphatic), 1597.37 (C = N stretch).

#### 5.1.6. Preparation of 2-(3-Anilino)-4-morpholino-5,6,7,8-tetrahydrobenzo[4,5]thieno[2,3-d]-pyrimidine (VIIa)

To a solution of the nitro derivative (**VIh**) (2.52 mmol, 1 equivalent) in DCM (100 ml), palladium-on-charcoal (10%) was added and then the mixture was stirred under H_2_ at room temperature, at 60 bar for 6 h using Parr hydrogynator apparatus after the flushing of reaction mixture 3 times with hydrogen. After removing the catalyst by filtration over filter aid (celite), the filtrate was evaporated under vacuum till complete dryness to give the titled compound (**VIIa**) which was purified through flash chromatography (gradient elution DCM: hexane = 9:1 till DCM) to give the titled compound (**VIIa**).

The titled compound (**VIIa**) was separated as off white powder (0.88 g, 95.2%), m.p. 181–183 °C; ^1^H-NMR (400 MHz, DMSO- d_6_): *δ* (ppm) 10.13 (s, 2H, NH_2_), 7.67 (s, 1H, ArH), 7.57 (d, *J* = 7.6 Hz, 1H, ArH), 7.12 (d, *J* = 7.9 Hz, 1H, ArH), 6.67 (d, *J* = 7.8 Hz, 1H, ArH), 3.81 (d, *J* = 5.1 Hz, 4H, morpholine), 3.42 (d, *J* = 6.2 Hz, 4H, morpholine), 3.01–2.79 (m, 4H, cyclohexyl CH_2_), 1.89 (d, *J* = 8.5 Hz, 2H, cyclohexyl CH_2_), 1.76 (d, *J* = 8.7 Hz, 2H, cyclohexyl CH_2_). ^13^C NMR (100 MHz, Chloroform-d_1_): *δ* (ppm) 169.29, 162.01, 157.67, 146.34, 138.98, 135.19, 129.30, 126.97, 119.66, 118.84, 117.04, 114.73, 66.69, 51.13, 26.69, 25.91, 23.05, 22.91. MS (Mwt = 366.48), *m/z* (% rel. int.): 366.22 (M^+^, 100%), 367.25 (M ^+1^+H, 25.76%). Anal. Calcd. for C_20_H_22_N_4_OS: C, 65.55; H, 6.05; N, 15.29; S, 8.75; Found: C, 65.81; H, 6.23; N, 15.48; S, 8.69. FT-IR (*ύ* max, cm^−1^): 3415.27–3336.21 (NH_2_ aromatic), 2954.4 (CH aliphatic).

#### 5.1.7. Preparation of 2-(4-Anilino)-4-morpholino-5,6,7,8-tetrahydrobenzo[4, 5]thieno[2,3-d]-pyrimidine (VIIb)

Compound (**VIi**) was dissolved in acetone (3 ml/mmol) and aqueous NaOH (0.5 N, 5equivalent). Excess sodium dithionite was added and the reaction was refluxed for 1 h. The completion of the reaction was monitored using TLC. When all nitro compound is consumed, the acetone was evaporated. The residue was taken up in ethyl acetate (50 ml) and washed with water, brine (3 × 30 ml). The organic layer was dried over anhydrous Na_2_SO_4_, filtered and evaporated in *vacuo* to give corresponding amine compound (**VIIb**) which was purified through flash chromatography (gradient elution DCM: hexane = 9:1 till DCM) to give the titled compound (**VIIb**).

The titled compound **VIIb** is yellow Powder (0.86 g, 93.04%), m.p. 206–208 °C; ^1^H-NMR (400 MHz, CDCl_3_-d_1_): *δ* (ppm) 10.15 (s, 2H, NH_2_), *δ* 8.49–8.31 (d, *J* = 7.8 Hz, 2H, ArH), 6.91–6.77 (d, *J* = 7.8 Hz, 2H, ArH), 3.98–3.86 (m, 4H, morpholine), 3.67–3.45 (m, 4H, morpholine), 2.89 (m, 4H, cyclohexyl CH_2_), 1.96 (m, 4H, cyclohexyl CH_2_). MS (Mwt = 366.48), *m/z* (% rel. int.): 366.28 (M^+^, 100%), 367.39 (M^+^+H, 26.35%). Anal. Calcd. for C_20_H_22_N_4_OS: C, 65.55; H, 6.05; N, 15.29; S, 8.75; Found: C, 65.78; H, 6.31; N, 15.54; S, 8.78

#### 5.1.8. Preparation of 2-(4-Acetamidophenyl)-4-morpholino-5,6,7,8-tetrahydrobenzo[4, 5] thieno-[2,3-d]pyrimidine (VIII)

To a solution of the amino derivatives (**VIIb**) (0.2 g, 0.545 mmol, 1 equivalent) in dry DCM (10 ml), acetic anhydride (0.06 ml, 0.655 mmol, 1.2 equivalent) was added drop wise to reaction solution in ice bath. Then the ice bath was removed and the reaction mixture was stirred under N_2_ at room temperature for 6 h. The titled compound was filtered, then purified through using flash chromatography (gradient elution hexane: ethyl acetate = 9:1 till 7:3) to give the titled compound (**VIII**) as dark yellow powder (0.19 g, 85.22%), m.p. 240–242 °C; ^1^H-NMR (400 MHz, DMSO-d_6_): *δ* (ppm) 10.15 (s, 1H, NH), 8.32 (d, *J* = 8.5 Hz, 2H, ArH), 7.71 (d, *J* = 8.6 Hz, 2H, ArH), 3.87–3.75 (m, 4H, morpholine), 3.44 (m, 4H, morpholine), 2.97–2.84 (m, 4H, cyclohexyl CH_2_), 2.08 (s, 3H, (CO)CH_3_), 1.89 (t, *J* = 5.6 Hz, 2H, cyclohexyl CH_2_), 1.76 (t, *J* = 5.8 Hz, 2H, cyclohexyl CH_2_). ^13^C NMR (100 MHz, DMSO-d_6_): *δ* (ppm) 169.02, 168.97, 162.09, 156.91, 141.68, 134.49, 132.31, 128.77, 127.77, 119.04, 66.33, 51.14, 26.58, 25.77, 24.57, 23.02, 22.74. MS (Mwt = 408.52), *m/z* (% rel. int.): 408.30 (M^+^, 100%), 409.33 (M^+^+H, 41.67%). Anal. Calcd. for C_22_H_24_N_4_O_2_S: C, 64.68; H, 5.92; N, 13.71; S, 7.85; Found: C, 64.90; H, 6.13; N, 13.98; S, 7.91. FT-IR (*ύ* max, cm^−1^): 3250.40 (NH amidic), 3123.75 (CH aromatic), 2933.14 (CH aliphatic), 1666.33 (C = O amide stretch).

#### 5.1.9. Preparation of 2-(4-(4-Methylbenzamido)phenyl)-4-morpholino-5,6,7,8-tetrahydrobenzo[4, 5]thieno[2,3-d]pyrimidine (IX)

To a solution of compound (**VIIb**) (0.2 g, 0.545 mmol, 1 equivalent) in dry DCM (10 ml), TEA and 4-methyl toloyl chloride (0.12 ml, 0.818 mmol, 1.5 equivalent) were added drop wise to reaction solution over 30 min in ice bath. Then the ice bath was removed and the reaction mixture was stirred at room temperature for 24 h. The titled compound (**IX**) was filtered, then purified through using flash chromatography (gradient elution hexane: ethyl acetate = 9:1 till 7:3) to give the titled compound (**IX**) as light beige powder (0.24 g, 90.75%), m.p. 250–252 °C; ^1^H-NMR (400 MHz, DMSO-d_6_): *δ* (ppm) 10.38 (s, 1H, NH), 8.38 (d, *J* = 8.5 Hz, 2H, ArH), 7.93 (t, *J* = 9.0 Hz, 4H, ArH), 7.36 (d, *J* = 7.8 Hz, 2H, ArH), 3.81 (d, *J* = 4.4 Hz, 4H, morpholine), 3.53 (d, *J* = 5.5 Hz, 4H, morpholine), 2.87 (d, *J* = 6.5 Hz, 4H, cyclohexyl CH_2_), 2.37 (s, 3H, CH_3_), 1.87 (s, 2H, cyclohexyl CH_2_), 1.84–1.67 (m, 2H, cyclohexyl CH_2_). ^13^C NMR (100 MHz, DMSO-d_6_): *δ* (ppm) 167.30, 166.00, 161.76, 156.21, 142.22, 142.05, 134.82, 132.28, 131.85, 129.7, 129.36, 128.74, 128.32, 120.36, 118.83, 66.37, 51.04, 26.69, 25.74, 23.01, 22.71, 21.49. MS (Mwt = 484.62), *m/z* (% rel. int.): 484.37 (M^+^, 100%), 485.38 (M^+^+H, 22.88%). Anal. Calcd. for C_28_H_28_N_4_O_2_S: C, 69.40; H, 5.82; N, 11.56; S, 6.62; Found: C, 69.67; H, 6.05; N, 11.79; S, 6.74. FT-IR (*ύ* max, cm^−1^): 3013 (CH aromatic), 2937.93 (CH aliphatic), 1672.40 (C = O amide stretch).

### 5.2. Biological evaluation

#### 5.2.1. *In vitro* anti-proliferative activity against 60 cell line panel

The NCI *in-vitro* anticancer screening is a two-stage process, beginning with the evaluation of all compounds against the full NCI 60 cell lines panel representing leukaemia, NSCLC, melanoma, colon cancer, CNS cancer, breast cancer, ovarian cancer, renal cancer and prostate cancer at a single dose of 10 µM. The output from the single-dose screen is reported as a mean graph (Supplementary material).

#### 5.2.2. *In vitro* cytotoxic activity assay against T-47D cancer cell line (IC50)

The *in vitro* cytotoxic activity assay against T-47D cancer cell line (IC_50_) assay for the most promising three compounds was performed at Nawah Scientific Inc., (Mokatam, Cairo, Egypt). The cell line IC_50_ activity against T-47D cell line was performed using SRB assay, where Assay Conditions used were discussed in (Supplementary).

#### 5.2.3. *In vitro* PI3K inhibitory assay

The *in vitro* enzyme inhibition assay for the synthesised compounds was performed at ThermoFisher Scientific (Madison, WI USA). The PI3K inhibitory activity against 3 isoforms was performed using Adapta™ Screening Protocol, where Kinase-Specific Assay Conditions used were discussed in (Supplementary).

### 5.3. Molecular modelling

#### 5.3.1. Field alignment study

The designed strategy involved the use of the *in-silico* field alignment technique provided by Cresset’s FieldAlign^®^ module in an attempt to illustrate the similarity of the molecular fields between the designed compounds and PI-103 (4) (as reference inhibitor for PI3Kα) and GDC-0941 (1) (as reference inhibitor for PI3Kβ and PI3Kγ). Database molecules were firstly imported from the saved pdf file. A conformation generation protocol was applied within Cresset’s FieldAlign^®^ to each molecule in the database before the alignment of our designed compounds to the reference molecules and the addition of molecular field points. The results of the alignment process were ranked in descending order and manually the best conformer was chosen.

#### 5.3.2. Molecular docking

A molecular docking study was performed using the C-DOCKER module of Accelrys Discovery Studio^®^ 2.5 (Accelrys Inc., San Diego, CA, USA) at the Faculty of Pharmacy, Ain Shams university, drug design laboratory.

##### 5.3.2.1. Preparation of protein

The X-ray crystal structures of PI3Kα co-crystallized with PI-103 (4) and PI3Kβ,γ co-crystallized with GDC-0941 (1) were downloaded from the Protein Data Bank at the Research Collaboration for Structural Bioinformatics (RCSB) website (www.rcsb.org) (PDB codes: 4L23 for PI3Kα, 2Y3A for PI3Kβ and 3DBS for PI3Kγ) and loaded in Accelrys discovery Studio^®^ 2.5. The preparation of the protein structure was performed using the default protein preparation tools built into the software. The missing hydrogen atoms were firstly added to the amino acid residues. Then, completing the missing residues and applying force field parameters were done using CHARMm forcefield. Steric clashes occurred as a result of hydrogens addition; thus, the whole protein structure was minimised through a minimisation protocol, but fixed constraints have been created on the heavy atoms to keep its 3D structure unchanged. Before running the docking process, identify all the proteins as the receptor and display the ligand-protein interaction to define the binding site then the ligand was deleted.

##### 5.3.2.2. Preparation of ligands before docking

The ligands’ structures were drawn using the sketching tools of Accelrys Discovery Studio^®^ 2.5. The preparation of the ligands was performed using the default Ligand preparation protocol of Accelrys Discovery Studio with adjusting the ionisation pH parameter to 7.4, hydrogen atoms addition, and without generation of isomer and tautomer.

##### 5.3.2.3. Docking of test set

C-DOCKER protocol was used to dock biologically active compounds into the binding site of each isoform. After running the protocol, ten docking poses were generated for each ligand docked and were thoroughly inspected for getting the best binding mode. The top-ranked poses were selected and investigated. The docking scores are displayed in energy terms (C-DOCKER Energy). The higher the score (in negative terms), the better the binding affinity.

## Data Availability

All data generated or analysed during this study are included in this published article in the main manuscript.
